# A computational process-tracing method for measuring people’s planning strategies and how they change over time

**DOI:** 10.3758/s13428-022-01789-5

**Published:** 2022-07-11

**Authors:** Yash Raj Jain, Frederick Callaway, Thomas L. Griffiths, Peter Dayan, Ruiqi He, Paul M. Krueger, Falk Lieder

**Affiliations:** 1 Max Planck Institute for Intelligent Systems, Tübingen, Germany; 2 Birla Institute of Technology and Science, Pilani, Hyderabad India; 3 Department of Psychology, Princeton University, Princeton, NJ USA; 4 Max Planck Institute for Biological Cybernetics, Tübingen, Germany; 5 Department of Computer Science, Princeton University, Princeton, NJ USA

**Keywords:** Process-tracing, Cognitive plasticity, Panning, Decision-making, Individual differences, Learning, Computational methods

## Abstract

One of the most unique and impressive feats of the human mind is its ability to discover and continuously refine its own cognitive strategies. Elucidating the underlying learning and adaptation mechanisms is very difficult because changes in cognitive strategies are not directly observable. One important domain in which strategies and mechanisms are studied is planning. To enable researchers to uncover how people learn how to plan, we offer a tutorial introduction to a recently developed process-tracing paradigm along with a new computational method for measuring the nature and development of a person’s planning strategies from the resulting process-tracing data. Our method allows researchers to reveal experience-driven changes in people’s choice of individual planning operations, planning strategies, strategy types, and the relative contributions of different decision systems. We validate our method on simulated and empirical data. On simulated data, its inferences about the strategies and the relative influence of different decision systems are accurate. When evaluated on human data generated using our process-tracing paradigm, our computational method correctly detects the plasticity-enhancing effect of feedback and the effect of the structure of the environment on people’s planning strategies. Together, these methods can be used to investigate the mechanisms of cognitive plasticity and to elucidate how people acquire complex cognitive skills such as planning and problem-solving. Importantly, our methods can also be used to measure individual differences in cognitive plasticity and examine how different types (pedagogical) interventions affect the acquisition of cognitive skills.

## Introduction

A remarkable feature of the human mind is its ability to improve itself continually. As helpless babies develop into mature adults, they not only acquire impressive perceptual and sensory-motor skills and knowledge about the world. They also acquire cognitive skills such as the abilities to perform mental arithmetic, plan, and problem-solve (van Lehn, 1996; Shrager & Siegler, 1998; Lieder & Griffiths, 2017; He et al., 2021; Jain et al., 2019). These abilities can be understood in terms of computational procedures that people perform on their mental representations of the external environment. Such computational procedures are known as *cognitive strategies*. Here, we focus on cognitive strategies for planning and refer to them as *planning strategies*. There are many different types of planning strategies that people can use. And as a person gains more experience they might switch from a less effective strategy to a more effective one. For instance, the first time a person plans a road trip they might start by thinking about which nearby location they might visit first, mentally simulating how good it would be to visit that location, then think about where they might go next, mentally simulating what it would be like to be there, and so on. By the time that this person plans their tenth road trip, she might start by mentally simulating especially attractive distant locations that the road should be designed to lead to. These two examples illustrate that people’s planning strategies draw on a shared set of elementary *planning operations* that mentally simulate states and actions but differ in what planning operation they perform under which conditions.

Developmental and learning-induced changes in how people think and decide are collectively known as *cognitive plasticity*. Just like the acquisition of perceptual skills (Hubel & Wiesel, 1970), the acquisition of cognitive skills requires specific experiences and practice (van Lehn, 1996; Ericsson et al., 1993). Despite initial research on how people acquire cognitive skills such as the abilities to perform mental arithmetic, plan, and problem-solve (van Lehn, 1996; Shrager & Siegler, 1998; Lieder & Griffiths, 2017; He et al., 2021; Jain et al., 2019), the underlying learning mechanisms are still largely unknown. Reverse-engineering how people discover effective cognitive strategies is very challenging. This is chiefly because it is impossible to observe directly people’s cognitive strategies or how people’s strategies and strategy choices change with experience – let alone the underlying learning mechanisms. Instead, cognitive plasticity has to be inferred from observable changes in behavior. This is difficult because any observed behavior could have been generated by many different cognitive mechanisms. This problem is pertinent to all areas of cognition.

**Fig. 1:**
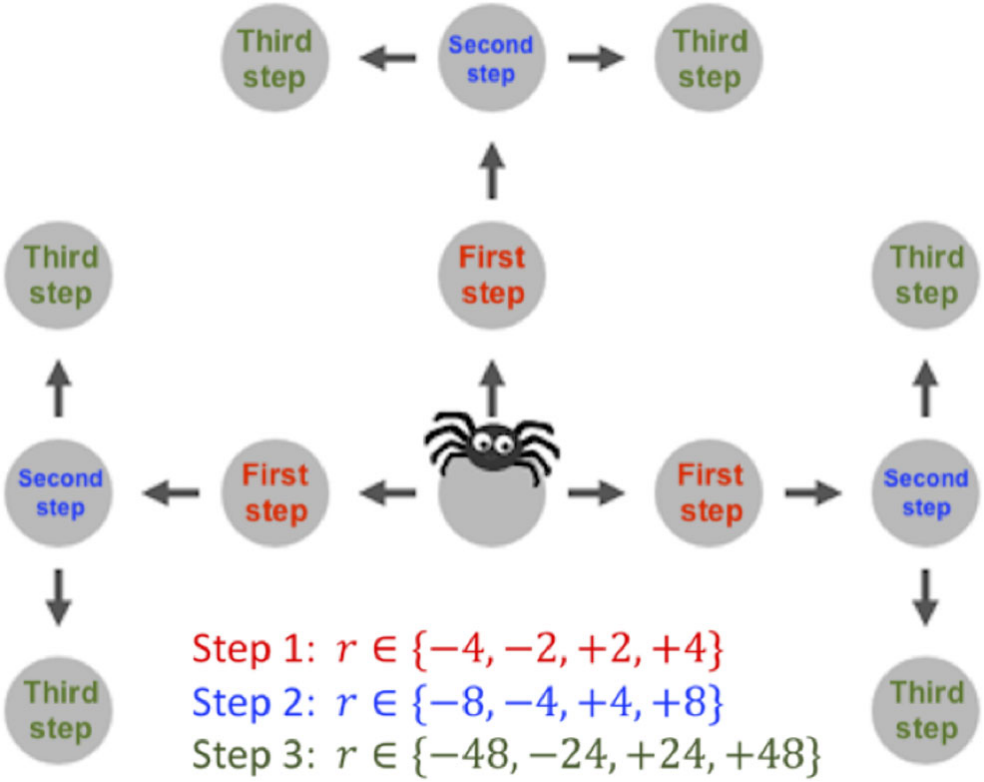
Illustration of the Mouselab-MDP paradigm. This figure shows a three-step planning task that can be created within the Mouselab-MDP paradigm. Here, the participant has to choose a series of three moves. Starting from the central location, the first decision is whether to move left, up, or right (Step 1); in each case there is only one option for the second move (Step 2), and then the spider can turn either left or right in the third step. Rewards are revealed by clicking, prior to selecting a path with the arrow keys. At each node each of the four possible rewards is equally likely to occur

**Fig. 2:**
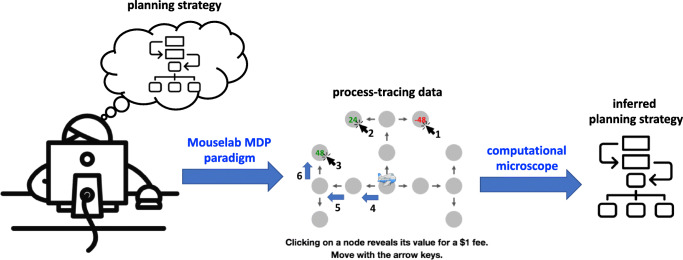
Illustration of the basic idea of measuring people’s planning strategies. The Mouselab MDP paradigm is a process-tracing method that utilizes mouse tracking to measure which pieces of information people inspect during planning and in which order they inspect them. The computational microscope is a model-based inference method that determines which of 79 different planning strategies the participant is most likely to have used on a given trial

We assume that each planning strategy performs a sequence of internal information gathering operations (Callaway et al., 2022b). Concretely, we assume that each of these planning operations mentally simulates what might happen if one took a particular action in a particular situation. We assume that the outcome of each simulation is the reward that the person expects the action to generate. Furthermore, we treat the mental simulation of each state-action pair as a separate planning operation. These assumptions make it possible to measure planning by externalizing the process of information gathering that would otherwise occur through memory recall and mental simulation (Callaway et al., 2017; Callaway et al., 2018; Callaway et al., 2022b). Building on this theory and a previous method for studying how people choose between alternatives with multiple attributes (Payne et al., 1993), we introduce a process-tracing paradigm for revealing the sequence of information gathering operations people perform during planning (see Fig. 1) and a computational method for inferring the underlying planning strategies (see Fig. 2). We will refer to these methods as the *Mouselab MDP* paradigm and our *computational microscope*.

**Fig. 3:**
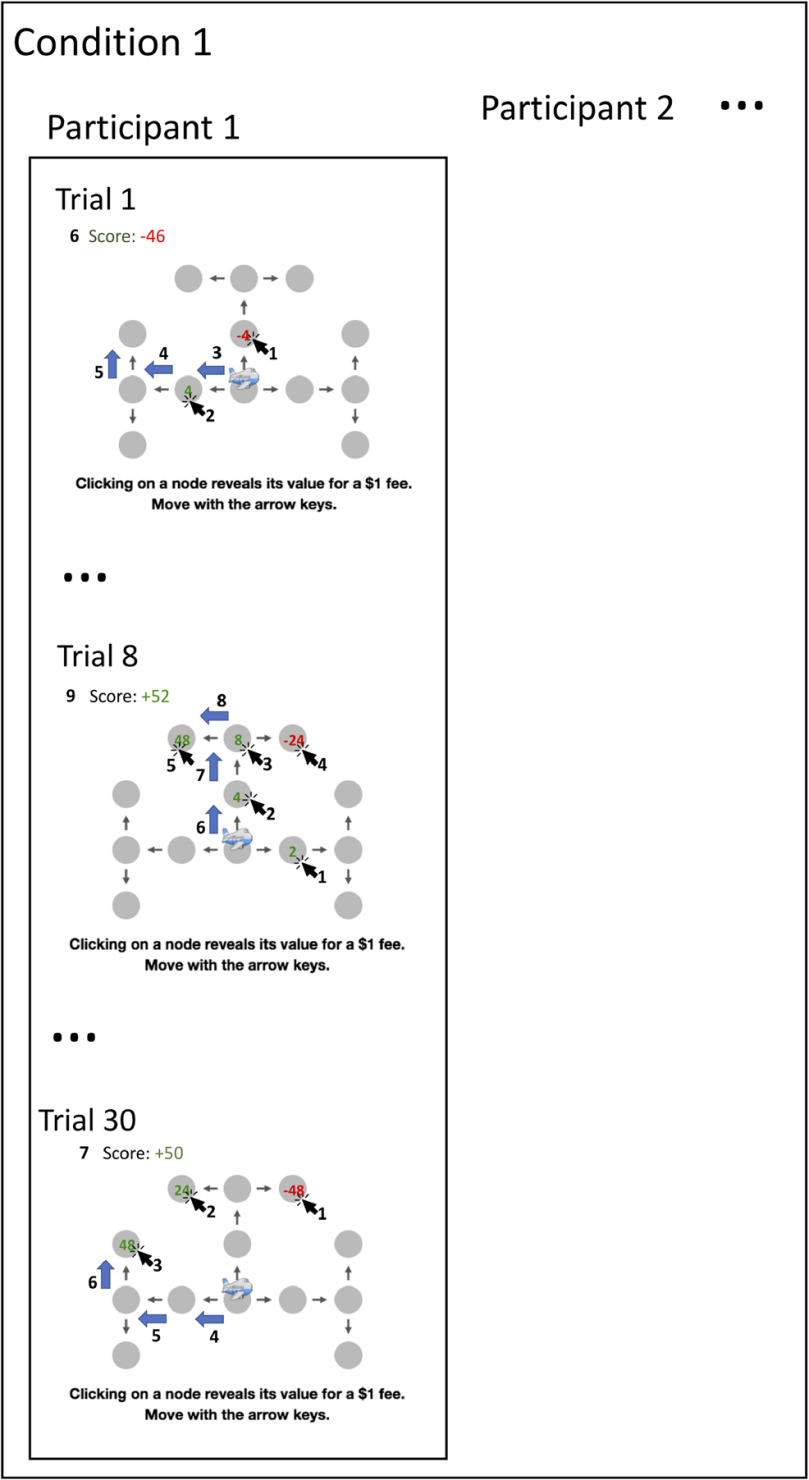
Illustration of the process-tracing data that can be collected with the Mouselab-MDP paradigm. The recorded interactions (clicks and moves) the participant made and the information the participant observed are enumerated in the order in which they occurred. In this example, the first participant started out with a short-sighted planning strategy and gradually discovered a more far-sighted one. On the first trial she made two clicks on immediate outcomes on their first trial and then selected a path. In the last trial the first participant inspected three final outcomes. The process-tracing data from the intermediate trials documents the participant’s transition between these two very different ways of planning

Our process-tracing method renders people’s behavior in a route planning task highly diagnostic of their planning strategies by

requiring them to click on locations they consider visiting to find out how costly or rewarding it would be to do so (see Figure 1). That is, when a person clicks on the state that they would get to by taking a certain action in a certain state, we treat it as an indication that they just performed the corresponding planning operation. The Mouselab-MDP paradigm poses people a series of planning problems (one in each trial). For each trial, it records the sequence of clicks (planning operations) that the participant performed, which information each click revealed, and the plan that the participant selected based on the resulting information (see Fig. 3). As Fig. 3 illustrates, this makes it possible to observe how the type of planning operations a person performs and the order in which she performs them change from each trial to the next. Our computational microscope uses the resulting process-tracing data to perform model-based inference on the trial-by-trial sequence of planning strategies the participant used to make his or her decisions. Together, these two methods allow researchers to specify a planning task and directly measure how people’s planning strategies change from one trial to the next (see Fig. 2). To facilitate adoption of the toolbox, we provide JavaScript and Python libraries for both components and a tutorial on how to use them. We hope that this toolbox will help researchers measure how people’s planning strategies change depending on their experience.

People changing their planning strategies in response to how well they worked is a prime example of what we call *metacognitive reinforcement learning* (Krueger et al., 2017; Lieder & Griffiths, 2017; Lieder et al., 2018c; Jain et al., 2019; He et al., 2021). Metacognitive reinforcement learning is set of mechanisms through which people learn when to perform which cognitive operations through trial and error. These mechanisms might play an important role in how people discover new cognitive strategies, adapt their strategies to the structure of their environment, and acquire cognitive skills (Lieder & Griffiths, 2017; Krueger et al., 2017; Jain et al., 2019; He et al., 2021).

Metacognitive learning is difficult to study because its effects and mechanisms cannot be observed directly. Throughout this article we will present a series of case studies to illustrate that our new computational method is useful for characterizing how people learn how to plan and elucidating metacognitive reinforcement learning more generally.

The plan for this paper is as follows: First, we summarize and illustrate the functionality offered by our toolbox for measuring how people learn how to plan and explain how it works. Next, we provide a practical step-by-step user’s guide on how to apply it. We then demonstrate the reliability and validity of the inferences of our computational microscope.

In closing, we discuss directions for future work enabled by the methodology introduced in this article.

## New methods for measuring how people learn how to plan

Planning, like all cognitive processes, cannot be observed directly but has to be inferred from observable behavior. This is generally an ill-posed problem. In previous work, researchers have inferred properties of human planning from the decisions participants ultimately made or asked participants to verbalize their planning process. However, many different planning strategies can lead to the same final decision, and introspective reports can be incomplete or inaccurate. In the 1970s researchers studying how people choose between multiple alternatives (e.g., apartments) based on several attributes (e.g., rent, size, location, etc.) faced a similar problem (Payne, 1976). To overcome this problem, Johnson et al., (1989) developed a *process-tracing* paradigm that elicits and records behavioral signatures of people’s decision strategies. Concretely, in the Mouselab paradigm (Payne et al., 1993), the alternatives’ attribute values are initially concealed and the participant can make clicks with their computer mouse to reveal one attribute value at a time. The Mouselab paradigm allows researchers to trace people’s decision strategies by recording which attributes of which alternatives people inspect in which order (Payne et al., 1993). While these behavioral signatures are still indirect measures of cognitive processes, and the means of observation might disturb the normal processes of decision-making, they do at least provide additional information about potential underlying decision strategies.

The Mouselab paradigm has enabled an extremely productive stream of research on the processes of multi-attribute decision-making (Payne et al., 1988; Ford et al., 1989; Payne et al., 1993; Schulte-Mecklenbeck et al., 2011; Schulte-Mecklenbeck et al., 2019). Here, we introduce two new methods that extend the process-tracing methodology from the domain of multi-attribute decision-making to the domain of planning. We start by describing a new process-tracing paradigm for measuring individual planning operations (Section 5). Measuring planning operations can yield valuable insights into how people plan (Callaway et al., 2017; Callaway et al., 2022b). But most research questions, such as how human planning compares to planning algorithms used in artificial intelligence, are not formulated at the level of individual planning operations but instead at the level of planning *strategies*.

Analyzing the data collected with our process-tracing paradigm suggested that people use a wide range of different planning strategies. We found that which strategy people use does not only depend on the structure of the environment (Callaway et al., 2018; Callaway et al., 2022b) but also on the participant’s learning history and individual differences. Concretely, we found that people may use as many as 79 different planning strategies across different environments and different points in time. These strategies prioritize different types of information, such immediate outcomes versus long-term consequences, highly uncertain outcomes, or outcomes following gains rather than losses, and they also differ in when they stop collecting more information (e.g., upon uncovering a path yielding a reward of at least $48). The resulting set of strategies includes variants of classic planning algorithms, such as breadth-first search, depth-first search, and best-first search, as well as several novel strategies, such as first identifying the best possible final outcome and then planning backward from it. The 79 planning strategies can be grouped into 13 different types, including goal-setting strategies with exhaustive backward planning, forward-planning strategies similar to breadth-first search, and forward planning strategies similar to best-first search (see Section A for a list of all strategies grouped by strategy type).

To make it possible for researchers to measure which strategies were used, we developed a computational method that leverages each participant’s process-tracing data to infer which strategy he or she used on the first trial, the second trial, the third trial, etc. We introduce this method in Section 5. The basic idea is to invert a probabilistic model of how the participant’s process-tracing data was generated by a series of planning strategies through Bayesian inference. This is a challenging methodological problem because people rarely execute any given strategy perfectly. We solve this problem by explicitly modeling the variability in the strategy that people use, in their execution of the strategy, and in the way the execution of the strategy manifests in their process-tracing data. In addition, we also model that there might be trials on which people don’t use any particular strategy or a strategy that is still unknown.

Our computational microscope can be applied to reveal people’s planning strategies in a wide range of different task environments. Used in combination, our two methods can be used to characterize the cognitive mechanisms of human planning, investigate how a person’s planning strategies evolve across trials, and uncover how planning strategies are affected by contextual factors and differ between individuals. Our methods support this research by providing trial-by-trial measurements of four aspects of human planning: the series of planning operations they performed, which of the 79 different planning strategies was the most likely source of those planning operations, which type of strategy it was, and how different types of previously postulated mechanisms (e.g., habits vs. Pavlovian mechanisms vs. reasoning) might have shaped a person’s planning on a given trial.

**Fig. 4:**
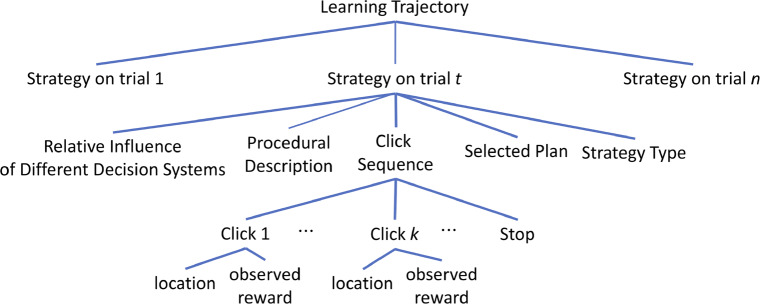
Illustration of the hierarchically nested information that our method provides about a participants planning throughout the *n* trials of a Mouselab-MDP experiment. The participant’s learning trajectory is characterized by the sequence of planning strategies that the participant used on trial 1, trial 2, ⋯, trial *n*, respectively. The strategy the participant used on a given trial is characterized by a procedural description, the general type of planning strategy it instantiates, the sequences of clicks it performed on that trial, the plan that they selected on that trial, and how the influences of different decision systems and other factors combine to generate that strategy. Each click sequence comprises a series of clicks. Each click is characterized by where the participant clicked and which information (reward) their click unveiled. Timing data is also available

Figure 4 summarizes the information that our computational microscope provides the user about how a given participant planned in a given Mouselab-MDP experiment. The following sections illustrate each of these functionalities in turn.

In this section we give a brief high-level overview of the functionality offered by our methods. The technical details are presented in the following section.

### Measuring individual planning operations with the Mouselab-MDP paradigm

To make individual planning operations measurable, we developed a process-tracing paradigm that externalizes people’s beliefs and planning operations as observable states and actions (Callaway et al., 2017). We refer to this paradigm as the Mouselab-MDP paradigm because it extends the approach of the Mouselab paradigm (Payne et al., 1993) to a general class of planning tasks known as Markov Decision Processes (MDPs) (Sutton and Barto, 2018). A Markov Decision Process comprises a series of decisions. Given the current state (e.g., location) the agent has to choose an action that, together with the current state, determines both an immediate reward and the next state. The task is to maximize the sum of all rewards over time. Inspired by the Mouselab paradigm (Payne et al., 1993), the *Mouselab-MDP* paradigm uses people’s mouse-clicking as a window into their planning. As illustrated in Fig. 1, this paradigm presents participants with a series of route planning problems. Each route planning problem is presented as a map where each location (the gray circles), harbors a gain or loss. These potential gains and losses are initially occluded, corresponding to a highly uncertain belief state. The participant can (expensively) reveal each location’s reward by clicking on it and paying a fee. This is similar to looking at a map to plan a road trip. Clicking on a circle corresponds to thinking about a potential destination, evaluating how enjoyable it would be to go there, or perhaps how costly it would be to go through there on the way to somewhere else, and then adjusting one’s assessment of candidate routes accordingly. The set of revealed rewards constitutes the state of the participant’s knowledge which we will refer to as the *belief state*. The tasks in this paradigm are designed such that each planning operation requires the participant to make a specific click and each click is the output of a specific planning operation. Participants can make as few or as many clicks as they like. After that the participant has to select a route through the environment using the arrow keys. For each location they visit, the corresponding reward is added to their score. The task is to maximize the money earned by traversing the environment minus the fees paid for collecting information.

The Mouselab-MDP paradigm can be used to create a wide range of environments that vary in size, layout (structure), and reward distribution. Figures 1, 7a-c, and 9 illustrate the variety of task environments that can be created with this paradigm. Several of the illustrative examples below and the experiments used to validate our methods are based on the simple three-step planning task shown in Fig. 1. Here, the participant can earn money by navigating a money-loving spider through a “web of cash”. There are six possible paths the participant can choose between. Each path comprises three steps, starts from the gray node in the center of the web, and proceeds along the arrows. In the first step, the spider can go left, up, or right. In the second step, it has to continue in that direction. In the third step, it can choose to either turn left or right. Each node that the spider might visit along the chosen path harbors a gain of up to *$*48 or loss of up to $-48. The player earns a monetary bonus proportional to the sum of the three rewards along the chosen path minus the fees they paid for clicking. In the beginning all gains and losses are concealed. The participant can uncover them for a fee of $1 per click. The participant can make as many or as few clicks as they like. Once they are done collecting information (planning), they start acting by moving the spider with the arrow keys. The participant receives the gain or loss at a given location if and only if they move the spider there. Clicking on a node only reveals the information which gain or loss they would receive if they moved to the inspected location but does not collect that reward. Furthermore, whether or not a node has been inspected has no effect on the reward the participant receives when the spider enters that location. Critically, in this particular three-step planning task, the variance of the potential rewards is smallest for the nodes that can be reached within one step, larger for the nodes that can be reached within two steps, and largest for the potential final destinations that are three steps away from the spider’s starting position at the center of the web (see Figure 1). This captures a common feature of real-world planning problems, namely that long-term outcomes are more important than short-term rewards.

The Mouselab-MDP paradigm can be used to elicit information about people’s planning operations at a level of detail which was inaccessible with previous behavioral paradigms. It makes it possible to measure which information people’s planning strategies consider in which order and how this depends on the information revealed by previous planning operations. Figure 3 illustrates the kind of process-tracing data that can be obtained with the Mouselab-MDP paradigm. The data from any given trial traces the strategy that an individual participant used to reach their decision on that trial. Taken together, the data from a series of trials traces how the participant’s decision strategy changed along with the observations and experienced rewards that preceded each change. Concretely, the example illustrated in Fig. 3 what the data might look for a participant who starts out with a myopic planning strategy and gradually discovers the optimal far-sighted goal-setting strategy.

### A computational microscope for inferring people’s planning strategies

The fine-grained information about the planning operations obtained from the Mouselab-MDP paradigm can be used to draw much richer inferences about how people plan and how the way they plan changes over time. However, the raw click sequences are difficult to analyze directly without sophisticated and typically theory-laden modeling tools. The computational microscope is a computational method that makes it possible to characterize how the participants of your experiment planned at the level of planning strategies, strategy types, and the contributions of different decision systems and other factors. In this section, we first give an overview of the computational microscope’s functionality. We then give a detailed account of how this functionality is implemented and close with an illustrative example of how the computational microscope can be used.

#### Overview of the computational microscope’s functionality

The computational microscope makes use of the information about people’s planning operations collected with the Mouselab-MDP process-tracing paradigm to help us better understand how people plan and how their planning changes over time. It makes it possible to infer which of 79 known planning strategies a participant used on a given trial from their clicks in the Mouselab-MDP paradigm. The set of 79 planning strategies includes the strategy that does not plan at all, a strategy that only inspects the immediate rewards, a strategy that inspects only the potential final outcomes and terminates planning once it discovers a large positive value, a variant of this strategy that plans backward from the preferred final outcome, search-based planning strategies (Russell & Norvig, 2016), such as breadth-first search (i.e. first explore nodes that are one step away, then explore nodes at are two steps away, and so on) and best-first search (i.e., explore nodes in decreasing order of the values of the paths they lie on), a strategy that explores all final nodes that are farthest away from the start node, and many others. For the hypothetical data set illustrated in Fig. 3, our computational microscope would likely infer that the participant started with the myopic planning strategy that terminates upon uncovering a positive value (Strategy 53 described in Section A) and eventually discover the optimal goal-setting strategy (Strategy 6 described in Section A).

In addition to fine-grained information about concrete planning strategies, the computational microscope also provides high-level information about which kind of planning strategy the person is using. Concretely, the microscope distinguishes between 13 types of planning strategies: four types of goal-setting strategies that explore potential final outcomes first, a strategy that explores immediate outcomes on the paths to the best final outcomes, a satisficing version of that strategy, forward-planning strategies (i.e strategies that start planning from nodes that are one step away from the start node) similar to Breadth First Search, middle-out planning (i.e the strategies that click the nodes in the middle of a path, then click the nodes that are nearest to the start node and then click nodes that are the farthest away), forward-planning strategies similar to Best First Search, local search strategies that focus on information about subtrees and next or previous steps along the paths that have received the most consideration so far, frugal planning strategies (i.e strategies that explore very little or not at all), myopic planning strategies (i.e. strategies that only explore nodes that are one step away from the start node) and a few other strategies that do not fit any of these categories. The four types of goal-setting strategies differ in how many potential goals they inspect (all vs. some), in how many and which earlier outcomes they inspect (all vs. some), and in when and how often they transition between inspecting goals versus earlier outcomes. For instance, goal-setting with exhaustive backward planning inspects all potential goals and all earlier outcomes. By contrast, frugal goal-setting strategies only explore some of the potential goals and none or only a small number of the earlier outcomes. Maximizing goal-setting with limited backward planning first identifies an optimal final outcome and then either terminates planning or inspects only the nodes on the path leading to the best final outcome. By contrast, maximizing goal-setting with exhaustive backward planning inspects the paths to all potential goals in the order of the goals’ rewards after having inspected all potential goals.

For the hypothetical data set illustrated in Fig. 3 our computational microscope would likely infer that the participant started with a frugal planning strategy and eventually discovered a maximizing goal-setting strategy with limited backward planning. The definitions of these strategy types are presented in Section A.

**Fig. 5:**
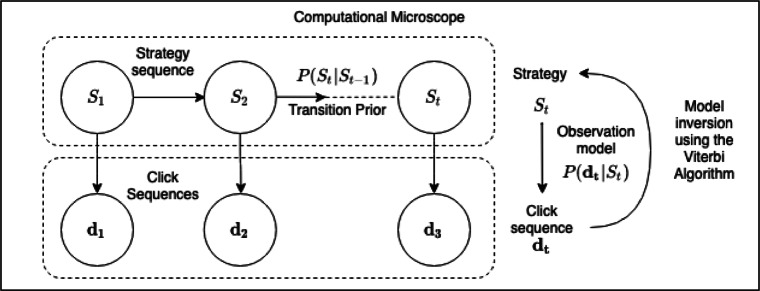
Overview of the computational microscope describing the Hidden Markov model that generates the observed process-tracing data as a graphical model

The computational microscope’s functionality is realized through model-based probabilistic inference. The model comprises three components: probabilistic models of 79 planning strategies, a probabilistic model of how planning strategies generate click sequences (*observation model*) and a probabilistic model of the sequence of planning strategies (*prior on strategy sequences*). As shown in Fig. 5, our method assumes that which planning strategy (*S*_*t*_) a participant uses can change from each trial (*t*) to the next but remains constant within each individual trial. In other words, we assume that exactly one planning strategy is used in each trial and that this strategy may be different from the one that was used in the previous trial and the one that will be used in the following trial. Furthermore, our method assumes that the strategies themselves do not change. Therefore, the computational microscope infers the trial-by-trial sequence of planning strategies that the participant used in the experiment (i.e., which strategy her or she used in the first trial of the experiment, which potentially different strategy he or she used in the second trial of the experiment, etc.). This sequence of planning strategies is inferred from the corresponding sequence of trial-by-trial click sequences (i.e., one click sequence for each trial). The basic idea is to find the sequence of planning strategies that is most likely to have generated the observed sequence of click sequences. The trial-by-trial changes in the relative influences of different decision systems and other factors can then be read off from the inferred strategy sequence because we make the simplifying assumption that way in which those factors interact to generate the behavior of a given strategy does not change over time. The computational microscope requires access to a set of planning strategies which generate the planning operations in a trial and models transitions among these strategies using a prior. We first describe how we formally model the planning strategies. We then describe the generative model of clicks (planning operations) given a strategy and then discuss how the computational microscope performs model inversion by taking into consideration information about participants’ clicks obtained from the Mouselab-MDP and the prior on strategy sequences to make inferences about the most likely sequence of strategies that might have generated the data. Obtaining the most likely sequence of strategies also gives us information about the strategy types and the temporal evolution of relative influence of decision systems (see Section 5).

#### Modeling planning strategies

To make it possible to extract interpretable strategies from the raw click sequences, we formulated a set of 79 planning strategies ($\mathcal {S}$) through a data driven methodology. Concretely, we manually inspecting the process-tracing data from an experiment in which participants completed 31 trials of the 3-step planning task illustrated in Fig. 1 (for description, see Appendix A.1). We visually inspected this data one click sequence at a time. Each time, we checked whether the current click sequence could be an instance of an already identified strategy. When this was not the case, we manually added an additional strategy to account for this new pattern. We then proceeded to the next click sequence and repeated the same procedure. If there was no apparent pattern, we identified it as an instance of a strategy that clicks randomly. We continued this process until our strategies were able to account for all click sequences of every participant who participated in the experiment described in Appendix A.1.

We modelled each of these planning strategies as a stochastic procedure that generates a sequence of planning operations (clicks). That is, a planning strategy specifies a probability distribution over what the first click might be and conditional probability distributions over what each subsequent click might be depending on which clicks were made previously and which rewards they revealed. For instance, the best-first search strategy distributes the probability of the first click evenly among the immediate outcomes and concentrates the probability of subsequent clicks on proximal outcomes that follow the best immediate reward(s). Furthermore, the planning strategy also specifies the conditional probability to terminate planning and select an action based on the information that has been revealed so far. For instance, for many of our planning strategies, the probability of terminating planning increases with the sum of the rewards of the best path that has been identified so far. As detailed in the next section, each planning strategy (*s*) entails a probability distribution (*P*) over which process tracing data (**d**) might be observed if a participant used that strategy (*P*(**d**|*s*)). Different strategies differ in which planning operations they perform first, in how they use the revealed information to select the subsequent planning operations, and in when they terminate planning. We model each sequence of planning operations a participant performed from the beginning of a trial to the end of that trial as the manifestation of a single strategy.^1^

According to our model, all strategies are probabilistic in the sense that they randomly select between all functionally equivalent planning operations that are consistent with what the strategy does in the current step. For instance, when the first step of a strategy is to inspect immediate outcomes until it uncovers a positive value, then our model assumes that the strategy chooses uniformly at random between all planning operations that inspect an uninspected immediate outcome. For more details about the strategies, please see Appendix A.4.

We found that, collectively, the 79 planning strategies can capture people’s click sequences much better than the random strategy. Concretely, we found that, on average, each click made by a participant is 3 to 6 times as likely under the best fitting strategy than under the random strategy. That is, for the environment with increasing variance, the maximum likelihood estimate of people’s strategies achieve an average click likelihood of 0.38 whereas the random strategy achieves an average click likelihood of only 0.10. For the environment with constant variance (Fig. 7b), the average per click likelihood is 0.50 whereas it is 0.09 for the random strategy. For the environment with decreasing variance (Fig. 7a), the average per click likelihood is 0.37 whereas it is 0.08 for the random strategy. And finally, for the environment used in the transfer task (Fig. 7c), the average per click likelihood is 0.19 whereas it is 0.03 for the random strategy.

#### Modeling how strategy sequences generate process-tracing data

To develop an efficient computational method for inferring the temporal evolution of people’s planning strategies, we make the simplifying assumption that the trial-by-trial sequence of peoples’ cognitive strategies (*S*_1_,*S*_2_,⋯ ,*S*_*T*_) forms a Markov chain whose hidden states emit the observed process tracing data collected on each trial (**d**_1_,⋯ ,**d**_*T*_). This hidden Markov model requires additional methodological assumptions about i) how cognitive strategies manifest in process-tracing data, ii) the set of cognitive mechanisms that can be learned (defined in Section 5), and iii) the nature and amount of cognitive plasticity that might occur. The following paragraphs detail our assumptions about the components i) and iii) in turn.

##### Observation model

To plan in the Mouselab-MDP paradigm participants have to gather information by making a sequence of clicks. Our observation model thus specifies the probability of observing a sequence of clicks *d*_*t*_ on trial *t* if the strategy was *S*_*t*_ (i.e., *P*(*d*_*t*_|*S*_*t*_)).

To achieve this, we quantify each planning strategy’s propensity to generate a click *c* (or stop collecting information) given the already observed rewards encoded in belief state *b* by a weighted sum of 51 features (*f*_1_(*b*,*c*),⋯ , *f*_51_(*b*,*c*)). The features describe the click *c* relative to this information (e.g., by the value of the largest reward that can be collected from the inspected location) and in terms of the action it gathers information about (e.g., whether it pertains to the first, second, or third step). A detailed description of the features and strategies is available in Appendix A.6.

The *depth* feature, for instance, describes each click by how many steps into the future it looks. The features and weights jointly determine the strategy’s propensity to make click *c* in belief state *b* according to

1 $$ P(\mathbf{d_{t}}|S_{t})=\prod\limits_{i=1}^{|\mathbf{d_{t}}|} \frac{\exp\left( \frac{1}{\tau} {\sum}_{k=1}^{|w^{(S)}|} w_{k}^{(S)} f_{k}^{(S)}(c_{t,i},b_{t,i}) \right)}{{\sum}_{c \in \mathcal{C}_{b_{t}}} \exp\left( \frac{1}{\tau} {\sum}_{k=1}^{|w^{(S)}|} w_{k}^{(S)} f_{k}^{(S)}(c,b_{t,i}) \right)} , $$

where *d*_*t*,*i*_ is the i^th^ click the participant made on trial *t* (or the decision to stop clicking and take action), the decision temperature *τ* was considered as a hyperparameter which was set by the inference procedure, and *w*^(*S*)^ is the weight vector of strategy *S*. According to this probabilistic soft-max model, all clicks are possible under each strategy in each situation but their probability is higher the better they are aligned with the strategy.

The strategies differ in how much information they consider (ranging from none to all to exploring all the nodes), which information they focus on, and in the order in which they collect it. Building on the observation model in Eq. 1, we represent each strategy by a weight vector $\mathbf {w}=\left (w_{1},\cdots ,w_{51} \right )$ that specifies the strategy’s preference for features such as more vs. less planning, exploring nodes with more uncertainty vs. less, considering immediate vs. long-term consequences, satisficing vs. maximizing, avoiding losses (cf. Huys et al.,, 2012), exploring paths that have a larger number of explored nodes, exploring nodes that are related to already observed nodes such as the ancestor nodes, successor nodes and siblings, and other desiderata. These weights are computed by generating data by simulating which clicks each strategy would make and then fitting the weights in Eq. 1 using Maximum Likelihood Estimation (MLE). These weights span a high-dimensional continuous space with many intermediate strategies and mixtures of strategies. Cognitive plasticity could be measured by tracking how those weights change over time. But this would be a very difficult ill-defined inference problem whose solution would depend on our somewhat arbitrary choice of features. As a first approximation, our method therefore simplifies the problem of measuring cognitive plasticity to inferring a time-series of discrete strategies. A detailed description of the features used in the observation model can be found in Appendix 5

##### Prior on strategy sequences

Inferring a strategy from a single click sequence could be unreliable. To smooth out its inferences, our method therefore exploits temporal dependencies between subsequent strategies by using a probabilistic model of strategy sequences.

Transitions from one strategy to the next can be grouped into three types: repetitions, gradual changes, and abrupt changes. While most neuroscientific and reinforcement-learning perspectives emphasize gradual learning (e.g., Hebb, 1949; Mercado, 2008; Lieder et al.,, 2018c), others suggest that animals change their strategy abruptly when they detect a change in the environment (Gershman et al., 2010). Symbolic models and stage theories of cognitive development also assume abrupt changes (e.g., Piaget, 1971; Shrager & Siegler, 1998), and it seems plausible that both types of mechanisms might coexist.

We considered three kinds of priors on the strategy transitions: gradual, abrupt and a combination of gradual and abrupt transitions. We did not find any significant relationship between the probability of transition from one strategy to the next and the distance between the strategies (see Appendix A.2.1). We found that the frequency of a transition from a strategy to itself was more likely than a transition from a strategy to some other strategy (*t*(975) = 7.55, *p* < 0.0001,BF > 1000). Model selection using either AIC (Akaike, 1974) or BIC (Schwarz & et al. 1978) values computed using the likelihood values of the maximum likelihood estimate of the strategy sequence also revealed the abrupt prior to be the best performing. Therefore, we use the abrupt prior for all our inferences. The gradual and the mixed priors are described in Section 5.

The *abrupt changes prior* assumes that transitions are either repetitions or jumps.

2 $$ \begin{array}{@{}rcl@{}} &&P(S_{t+1}=s|S_t,m_{\text{abrupt}}) = \\ &&\ \ p_{\text{stay}}\mathbb{I}(S_{t+1}=S_t)+(1-p_{\text{stay}})\frac{\mathbb{I}(s \neq S_t)}{|\mathcal{S}|-1}, \end{array} $$

where $\mathcal {S}$ is the set of strategies, $|\mathcal {S}|$ is the number of strategies and *p*_*stay*_ is the probability of strategy repetitions.

We model the probability of the first strategy as a uniform distribution over the space of decision strategies (i.e., $P(S_{1})=\frac {1}{|\mathcal {S}|}$).

Together with the observation model and the strategy space described above, the prior defines a generative model of a participant’s process tracing data **d**; this model has the following form:

3 $$ \mathit{P}(\mathbf{d},S_{1},\cdots,\mathit{S}_{T}) = \frac{1}{|\mathcal{S}|} \prod\limits_{t=2}^{T} \mathit{P}(S_{t}| S_{t-1} | m_{\text{abrupt}}) \mathit{P}(\mathbf{d_{t}}|S_{t}).  $$

Inverting this model gives rise to a computational method for measuring an important aspect of cognitive plasticity.

#### Inferring strategy sequence by model inversion

Our model describes how the sequences of planning strategies a participant uses across the different trials of the experiment manifests in their process-tracing data. To measure this sequence of planning strategies, we have to reason backwards from the process tracing data **d** to the unobservable cognitive strategies *S*_1_,⋯ ,*S*_*T*_ that generated it. To achieve this, we first model the generation of process-tracing data using a Hidden Markov Model with the 79 planning strategies as the possible values of its latent states and the prior *m* +_abrupt_ as its transition prior. Having modelled how likely alternative strategies are to generate a given sequence of clicks, we can apply Bayes theorem to compute how likely a person is to have used different planning strategies given the clicks that they have made. More concretely, the computational microscope computes the sequence of strategies *s*_1_,*s*_2_,⋯ ,*s*_*T*_ that is most likely to have given rise to the process-tracing data observed on the corresponding *T* trials (**d**_1_,**d**_2_,⋯ ,**d**_*T*_). This is achieved by applying the Viterbi algorithm (Forney, 1973) to compute the maximum a posteriori (MAP) estimate $\arg \max \limits _{s_{1},s_{2},\cdots ,s_{T}} P(s_{1},s_{2},\cdots , s_{T} | \mathbf {d}_{1},\mathbf {d}_{2},\cdots ,\mathbf {d}_{T})$ of the hidden sequence of planning strategies *S*_1_,⋯ ,*S*_*T*_ given the observed process tracing data **d**, the measurement model *m*_abrupt_, and the parameter (*p*_stay_ of Eq. 2 and the strategy temperature parameter *τ* of the observation model. This inference combines the likelihood that a possible strategy would generate an observed click sequence with how probable potential sequences of planning strategies are a priori. The prior probability of strategy sequences is assigned based on the knowledge that people are often somewhat more likely to repeat the strategy they used on the previous trial than to switch an arbitrary other strategy.

To estimate the model parameter *p*_stay_ we perform grid search with a resolution of 0.02 over *p*_stay_ ∈ [0,1]. The value of *τ* is set using 50 iterations of Bayesian Optimization, with the likelihood of MAP estimate of the click sequence as the objective it maximizes. We use the Tree-structured Parzen estimator approach to Bayesian Optimization implemented in the hyperopt Python package (Bergstra et al., 2013) for optimizing the parameter *τ*.

Inferring the hidden sequence of cognitive strategies in this way lets us see otherwise unobservable aspects of cognitive plasticity through the lens of a computational microscope.

#### Inference on strategy types and meta-control

To understand what types of strategies people use, we grouped our 79 strategies using hierarchical clustering on the distances between the strategies. Since the strategies are probabilistic, we defined the distance metric Δ(*s*_1_,*s*_2_) between strategy *s*_1_ and *s*_2_ as the Symmetrised Kullback-Leibler divergence

between the distributions of click sequences and belief states induced by strategies *s*_1_ and *s*_2_ respectively, that is

4 $$ \begin{array}{@{}rcl@{}} {\Delta}{ (s_1, s_2)} &=& \text{JD}\left[p(\mathbf{d}|s_1),p(\mathbf{d}|s_2)\right] \\ &=& \text{KL}\left[p(\mathbf{d}|s_1),p(\mathbf{d}|s_2)\right] \\&&+ \text{KL}\left[p(\mathbf{d}|s_2),p(\mathbf{d}|s_1)\right], \end{array} $$

and approximated it using Monte-Carlo integration.

Applying Ward’s hierarchical clustering method (Ward, 1963) to the resulting distances suggested 13 types of planning strategies described in Section 5.

As discussed in Section 5, we assume that people’s choice of planning operations is shaped by the interactions of multiple decision systems and other factors. To measure the contribution of each factor in a strategy, we first assigned each feature to one of the decision systems. Then, for each decision system, we added the weights of the features which belonged to that decision system if the feature represented an increase in that decision system and subtracted it if it represented a decrease in that decision system to give us a weight *w*_*ds*_ for a decision system. The relative influence of the decision system on a strategy is measured by:

5 $$ \begin{array}{@{}rcl@{}} \text{RI}_{ds} = \frac{|w_{ds}|}{\sum\limits_{ds \in D}{|w_{ds}|}} , \end{array} $$

where *D* is the set of all decision systems.

#### An example of applying the computational microscope

To illustrate the functionality of our computational microscope, we applied it to data from an experiment evaluating intelligent tutors that teach people effective planning strategies (i.e., the experiment described in Appendix A.1). In this experiment participants practiced planning in the three-step decision task illustrated in Fig. 1 (see Section 5) for 10 trials (training block) and were then tested on 20 more trials of the same task (test block). Participants in the experimental conditions received two different types of feedback during the training block. Participants in the control condition received no feedback.

Table 1 lists all strategies that people used on at least 2% of the trials ordered by strategy type and frequency. As can be seen, the most common strategy types were maximizing goal-setting with limited backward planning, frugal planning, local search, myopic planning, frugal goal-setting, and other miscellaneous strategies that don’t belong to any other strategy type. These 6 types of strategies jointly accounted for 96.5% of all strategies that people used in this environment. For more information about these strategy types and the corresponding planning strategies, please see Appendix A.4.

**Table 1:** Summary of the planning strategies that people used most frequently in the environment illustrated in Fig. 1

Strategy type	Strategy	Used on __% of trials	Used by __% of people	People who used this strategy (type) used it on __% of trials
Maximizing goal-setting with limited backward planning	**50.4%**	**68.8%**	**69.7%**	
	Random search for best possible final outcome	36.6%	55.1%	63.1%
	Consecutive second maximum	6.3%	17.6%	33.8%
	Extra planning after observing second best outcome	2.2%	10.2%	20.6%
Frugal planning		**14.8%**	**34.1%**	**41.2%**
	No planning	13.2%	26.1%	47.8%
Miscellaneous strategies		**11.3%**	**43.8%**	**24.6%**
	Explore immediate outcomes of second best outcomes	2.2%	11.4%	18.0%
Local Search		**7.4%**	**27.3%**	**25.8%**
	Satisficing Depth First Search	3.6%	14.2%	24.3%
	Priority to explored immediate ancestors	2.1%	8.0%	25.0%
Myopic planning		**6.5%**	**29.0%**	**21.2%**
	Explore all immediate outcomes with satisficing	2.1%	9.7%	20.4%
	Explore all immediate outcomes	2.1%	9.1%	22.3%
Frugal goal-setting		**6.1%**	**29.0%**	**20.0%**
	Goal-setting with positive satisficing	2.1%	8.0%	24.5%

### Measuring the relative contributions of different decision systems and other factors

How people plan is shaped by the interaction of multiple different types of mechanisms throughout the decision-making process (van der Meer et al.,, 2012; Huys et al.,, 2012, 2015; Dolan & Dayan, 2013; Cushman & Morris, 2015; Keramati et al.,, 2016; Daw, 2018). In most real-life decisions it is infeasible or unwise to consider all possible sequences of actions, states, and outcomes. To decide which alternatives to consider and which ones to ignore, the model-based system relies on the recommendations of simpler mechanisms such as Pavlovian impulses (Huys et al., 2012), value estimates learned through model-free reinforcement learning (Cushman & Morris, 2015), and simple heuristics (Huys et al., 2015). Furthermore, previous findings indicate the existence of an additional decision system that is specialized for deciding between continuing to gather information (e.g., by foraging) versus acting on the information that is already available (Rushworth et al., 2012). Since deciding how to plan is like foraging for information, the decision when to stop planning might also be made separately from the decision how to plan. This decision can be made by determining whether the best plan identified so far is already good enough (satisficing) or other stopping criteria. In addition, people are also known to engage in metareasoning (Ackerman & Thompson, 2017; Griffiths et al., 2019) – that is reasoning about reasoning – to figure out what is the best way to figure out what to do. Furthermore, all else being equal, the way in which people decide seems to follow the law of least mental effort (Patzelt et al., 2019; Balle, 2002; Kool et al., 2010), that is people seek to avoid mental effort.

We assume that all of these factors simultaneously influence how a person selects his or her individual planning operations while making a single decision (Keramati et al.,, 2016; Huys et al.,, 2012, 2015; Daw, 2018). To measure the relative contributions of these different types of factors to each of the 79 planning strategies, we divided the features whose weights determine the strategies’ preferences for alternative planning operations into five categories: *Pavlovian*, *model-free values and heuristics*, *model-based metareasoning*, *mental effort avoidance*, and *satisficing and stopping criteria*.

The *Pavlovian* features report how attractive or repelling it is to think about a state based on the rewards and losses that precede or follow it. The category *model-free values and heuristics* includes structural and relational features of state-action pairs that people might come to associate with rewarded versus unrewarded planning operations. The features in the category *model-based metareasoning* are derived from a model of how alternative planning operations reduce the decision maker’s uncertainty about which plan is best. The category *mental-effort avoidance* includes a single feature that distinguishes between performing a planning operation (more mental effort) versus acting without further planning (less mental effort). The features in the category *satisficing and stopping criteria* describe conditions under which specific stopping rules would terminate planning, such as whether there is a path whose expected return exceeds $48 which is an instance of satisficing (Simon, 1955). For a detailed definition of these categories in terms of the constituent features see Appendix A.6. To measure the relative influence of these five types of factors on how a person planned on a given trial, we first sum up the weights that the inferred strategy assigns to features of this type to get a total weight for the type and then normalize its absolute value by the sum of absolute values of total weights of all types. Performing this calculation separately for first, second, third, ⋯, last trial allows us to track how the relative influence of different decision systems (i.e., the model-based system, the Pavlovian system, and model-free systems) and other factors (i.e., mental effort avoidance and stopping criteria) changes as people learn how to plan.

For the hypothetical data set illustrated in Fig. 3 our computational microscope would likely infer that the participant started out relying primarily on structural features (a sub-category of model-free values and heuristics), satisficing features, and mental effort avoidance. Furthermore, it would most likely infer that the participant then transitioned to relying increasingly more on model-based metareasoning features.

### Measuring cognitive plasticity

**Fig. 6:**
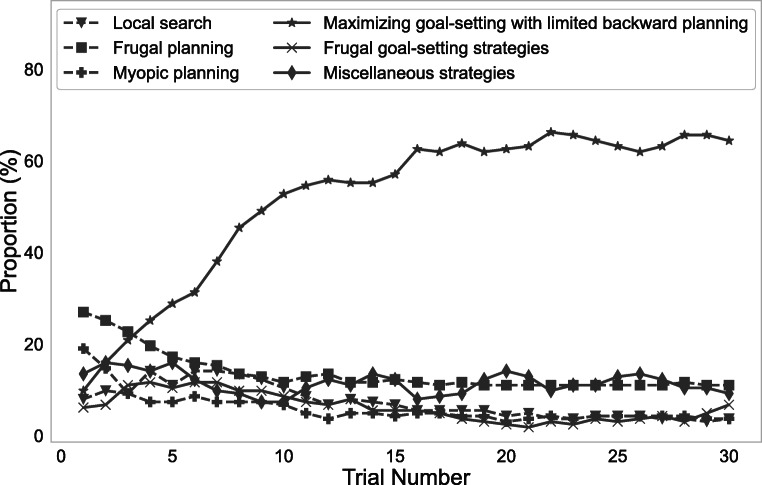
Measured time course of frequencies of strategy types in the experiment described in Appendix A.1.

Our method makes it possible to measure how people’s approach to planning changes at multiple levels of resolution across time scales ranging from seconds to decades. It can resolve changes in people’s planning at the level of individual planning operations, planning strategies, strategy types, and the contributions of different decision systems and other factors. By default, our method’s temporal resolution is the amount of time that passes from one trial to the next. This makes it suitable for reverse-engineering the learning mechanisms through which people discover and continuously refine their planning strategies (Jain et al., 2019). It can also measure how people’s approach to planning evolves over longer time scales, such as blocks, sessions, years, and decades. This makes the computational microscope suitable for investigating how people learn how to plan and how they adapt their planning strategies to new environments. Figure 6 illustrates the computational microscope’s ability to reveal how people’s propensities towards different types of planning strategies evolve as they learn how to plan in the task illustrated in Fig. 1; to obtain these results we applied the computational microscope to the data from the control condition of the experiment described in Appendix A.1. The output of the computational microscope revealed that the strategies which explore the final outcomes first and terminate upon finding a high value became the most frequent strategy type. During this transition people shifted away from frugal planning strategies (i.e., strategies that explore only a few outcomes) which were the most common strategies at the start of the experiment along with the myopic planning strategies (strategies that explore immediate outcomes first). The miscellaneous strategies also decreased in frequency. The frequency of local search (i.e., the strategies that focus on information about subtrees or paths that have been explored the most so far) and frugal goal-setting strategies (i.e., strategies that start exploring from the final outcomes and only explore a few outcomes) initially became more frequent and then decreased again.

**Fig. 7:**
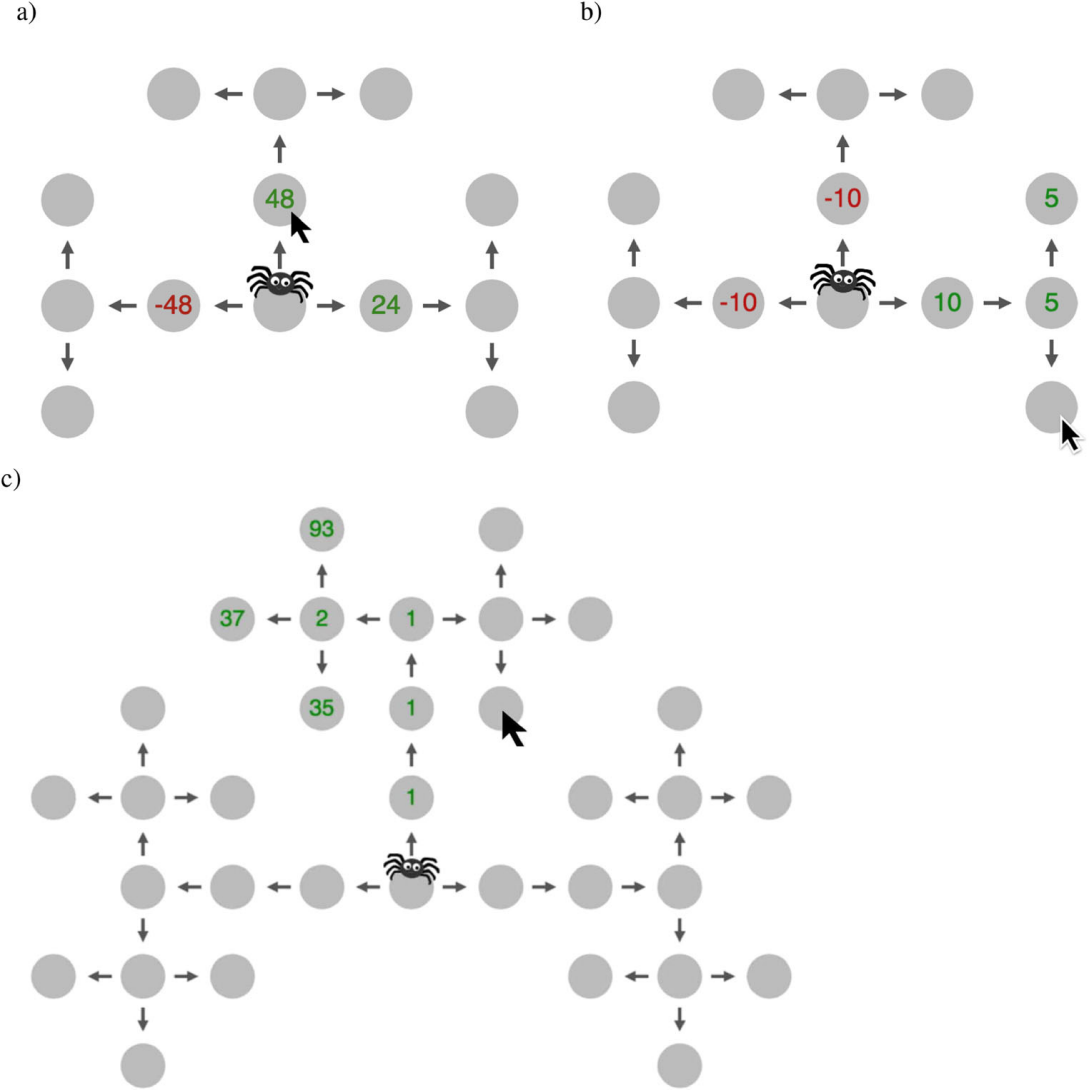
Illustration of the environment with decreasing variance (a), the environment with constant variance (b), and the five-step version of the environment with increasing variance (c). In the environment with decreasing variance, the rewards at the first, second, and third step are sampled uniformly at random from the sets {− 48,− 24,+ 24,+ 48}, {− 8,− 4,+ 4,+ 8}, and {− 4,− 2,+ 2,+ 4}, respectively. In the environment with constant variance, the rewards at all locations are independently sampled from the same uniform distribution over the set {− 10,− 5,+ 5,+ 10}. In the five-step planning task with increasing variance the rewards at steps 1 to 4 are drawn from normal distributions with mean 0 and standard deviation *σ*_1_ = 2^0^, *σ*_1_ = 2^1^, *σ*_1_ = 2^2^, and *σ*_1_ = 2^3^, respectively, and the reward at step 5 is drawn from a normal distribution with mean 0 and standard deviation *σ*_5_ = 2^5^

**Fig. 8:**
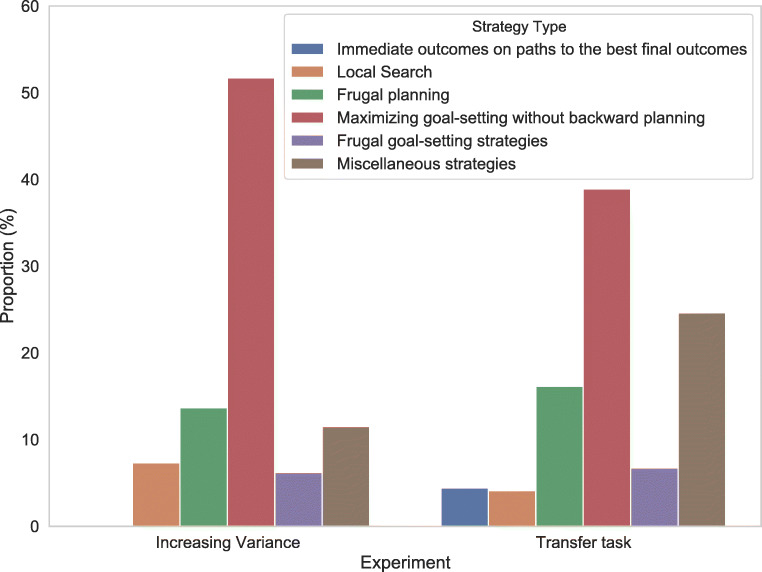
Comparison of frequencies of strategy types between the environment with increasing variance and transfer task. For a detailed description of the strategy types see Appendix A.4

In addition, the computational microscope can also be used to measure the transfer of learning from one task to another. Traditionally, transfer effects are established by demonstrating the training’s effect on people’s average performance in an untrained task. The computational microscope makes it possible to determine whether people transfer the specific strategies they learned in the training task to untrained tasks. To illustrate this, we applied the computational microscope to data from a transfer experiment in which participants practiced planning in a simple, small environment and were then tested on a larger and more complex environment. Concretely, the participants in the second experiment from Lieder (2018b) performed the five-step planning task illustrated in Fig. 7c after having practiced planning in the three-step planning task illustrated in Fig. 1 with optimal feedback (experimental condition) or without feedback (control condition). As shown in Fig. 8, the computational microscope revealed that participants from both conditions transferred the near-optimal goal-setting strategy they had learned in the three-step planning task to the five-step planning task.

Furthermore, our approach can also be used to characterize how people’s approach to planning changes across the lifespan (Das et al., 2019). Finally, our method can also be used to detect and compare the effects of (pedagogical) interventions on how people learn how to plan and to elucidate inter-individual differences in metacognitive learning (e.g., in psychiatric disorders).

## A step-by-step guide to measuring how people learn how to plan

Experimenters can make use of our paradigm and our computational microscope very easily. In this section, we provide a tutorial like introduction for running experiments with the Mouselab-MDP paradigm and applying the computational microscope on data generated using the Mouselab- MDP paradigm.

### A step-by-step guide to creating and running process-tracing experiments with the Mouselab-MDP paradigm

**Fig. 9:**
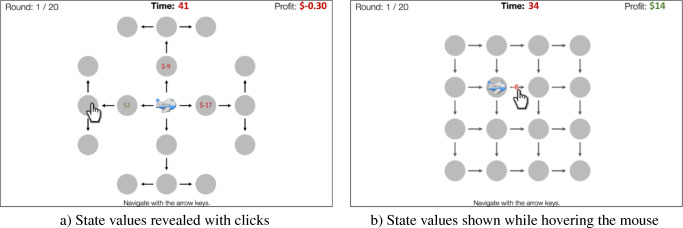
Two example paradigms created with the Mouselab-MDP plugin for JsPsych: a) Each state is labeled with the reward for reaching that state; these rewards become visible after they are clicked, with a $0.10 fee per click. b) The reward for making a transition is revealed only while the mouse is hovering over the corresponding arrow

Having motivated the paradigm, we briefly describe both the interface through which experimenters specify experiments, and the interface through which participants engage in the task. Two screenshots of the paradigm are shown in Fig. 9, and a live demo can be viewed at http://cocosci.princeton.edu/webexpt/mouselab-demo/. The code for Mouselab-MDP and an example of how to use it are available at https://github.com/RationalityEnhancement/Mouselab-MDP.

On each trial, an environment is conveyed by an intuitive visualization (see Fig. 9). Formally, each environment corresponds to a directed graph with states as nodes and actions as edges. The participant navigates through the graph using the keyboard, attempting to collect the maximal total reward. States or edges are annotated with the reward for reaching the state or taking the action. Crucially, these labels may not be visible when the trial begins. Rather, the participant may need to click or hover their mouse over a state or edge to see the associated reward. The timecourse of these information-gathering operations provides fine-grained information about the person’s planning strategy. Furthermore, our paradigm allows researchers to investigate how people negotiate the tradeoff between the cost of thinking and its benefits. This can be done by manipulating the cost of information gathering; for instance by charging participants a certain number of points per click.

With the Mouselab-MDP jsPsych plugin, experimenters can create a planning experiment by specifying the following critical components:

1. graph is a mapping *s*↦*A* from a state *s* to action contingencies *A*. Each action contingency is a mapping $a \mapsto (r, s^{\prime })$ from an action to a reward *r* and the next state $s^{\prime }$. The graph structure thereby specifies the actions *a* available in each state, as well as the reward *r* and resultant state $s^{\prime }$ associated with each action.
2. initial is the state in which the participant begins the trial.
3. layout is a mapping *s*↦(*x*,*y*) that specifies the location of each node on the screen.

Specifying only these settings will result in a graph with rewards shown on the edges between nodes and no labels on the states.

To take advantage of additional Mouselab features, the user must specify at least one of the following optional properties:

1. stateLabels is a mapping *s*↦*ℓ* that specifies the labels to be shown on each state.
2. stateDisplay∈ { ‘never’, ‘hover’, ‘click’, ‘always’ } specifies when state labels are displayed. When set to ‘click’, clicking on the state causes the label to appear and remain visible until the end of the trial. The optional parameter stateClickCost specifies the cost (a negative number) for clicking on a single state. When set to ‘hover’, the label appears only while the mouse is hovering over the associated edge. There is no cost for this option because the participant’s mouse might pass over an edge by accident.
3. edgeLabels is analagous to stateLabels, except that it defaults to the rewards associated with each edge.
4. edgeDisplay is analagous to stateDisplay. edgeClickCost specifies the cost.

Using this concise yet flexible plugin, various state-transition and reward structures can be displayed automatically. This allows experimenters to quickly create a large number of highly variable stimuli. Our plugin thereby enables experimenters with only basic knowledge of JavaScript to create a wide range of qualitatively novel experiments that can be run online with crowd-sourcing services such as Amazon Mechanical Turk.

### Step-by-step guide on using the computational microscope

Given a data set collected with the Mouselab-MDP paradigm with uniform click costs and no edge rewards, our computational microscope can be used to obtain a detailed analysis of how the participants learned how to plan without any additional programming . Here, we provide a step-by-step guide to applying the computational microscope. To help users get started with the computational microscope without having to collect data first, the computational microscope comes with data from four experiments using the tasks illustrated in Figs. 1 and 7a-c, respectively. The computational microscope provides information about the strategy sequence, the amount of noise in the application of the strategy, the sequence of strategy types and the change in the relative frequency of decision systems. The computational microscope requires git and Python3 to be installed on the user’s machine. The following steps describe how to apply the computational microscope to a data set and the output it provides.

1. Access data sets and the source code of the computational microscope by cloning the corresponding github repository using the command:
git clone https://github.com/RationalityEnhancement/ComputationalMicroscope.git
  The repository includes four data sets that are contained in the folder data/human/. For a detailed description of these data sets, see Table 2
2. Navigate to src/ and install the package requirements running the following command in the cloned repository’s root directory:
pip3 install -r requirements.txt
3. Apply the computational microscope on any of the 4 data sets described in Table 2 using the following command:
python3 infer_sequences.py <dataset> <block> <condition>
  The values that the parameters in the above command take can be found out by using the command:
python3 infer_sequences.py help
  Here, the parameters <dataset>, <block> and <condition> define the name of the dataset, the block of the experiment which generated the dataset, and the condition of the experiment, the computational microscope is to be run on. Upon successful completion, a dictionary with the participant IDs as keys and the strategy sequences as its values are stored as a pickle file in the path "results/inferred_sequences/<dataset> _<block>_<condition>_strategies.pkl" and the corresponding noise parameter values, in the same format, are stored in "results/inferred_sequences/<dataset> _<block>_<condition>_temperatures.pkl".
  For example, to run the computational microscope on the test block of the dataset with increasing variance for participants who belong to the condition without feedback, run the following command:
python3 infer_sequences.pyincreasing_variance train none
4. Analyze the generated sequences by running the command:
python3 analyze_sequences.py <dataset><block> <condition>
  This command produces plots of the trial-by-trial changes in the frequencies of the top-5 strategies and strategy types, and in the influence of different decision systems and other factors. It integrates the data from all participants into the plots in the "results/<dataset>_plots" directory.

**Table 2:** Datasets included in the computational microscope repository

Dataset	increasing_variance (v1.0)	decreasing_variance (c2.1)	constant_variance (c1.1)	transfer_task (T1.1)
Blocks	training, test	training, test	training, test	pre-training, training, test
Condition	meta, action, none	none	none	none
Description	This dataset contains process-tracing data from the experiment with the environment shown in Fig. 1. The experiment consisted of 10 training trials and 20 test trials had three conditions that determined the kind of feedback that was provided to the participants.	This dataset contains process-tracing data from the experiment with the environment shown in Fig. 7a. The experiment consisted of 30 training trials and 30 test trials only had a single condition.	This dataset contains process-tracing data from the experiment with the environment shown in Fig. 7b. The experiment consisted of 30 training trials and 30 test trials only had a single condition.	This dataset contains process-tracing data from the experiment with the environment shown in Fig. 7c. The experiment consisted of 1 pre-training trial, 10 training trials 20 test trials only had a single condition.
Reference	Appendix A.1	Callaway et al., (2018)	Callaway et al., (2018)	Lieder (2018a)

The value in brackets references the experiment number in the code

**Fig. 10:**
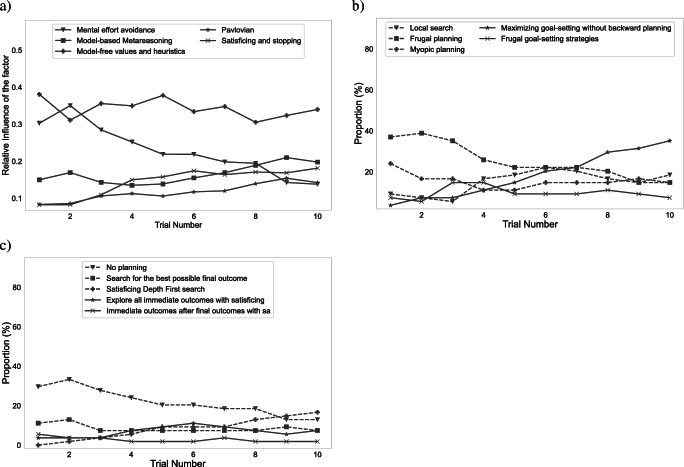
Generated analysis plots for training block of the no feedback condition of the increasing variance data set. **a** Influence of different decision systems and other factors. **b** Trial-wise changes in strategy type frequencies. **c** Trial-wise changes in strategy frequencies
  For example, the following command generates the plots shown in Fig. 10.
python3 analyze_sequences.pyincreasing_variance test none

The computational microscope, in its current implementation, can be applied to task structures that are symmetric and do not have cycles. But the general approach described in this article works for arbitrary environments. The implementation and a detailed tutorial on applying the computational microscope to a custom dataset are available at https://github.com/RationalityEnhancement/ComputationalMicroscope.

## Does it work?

To test whether using the computational microscope in conjunction with the Mouselab-MDP paradigm is a reliable way to measure how people plan, we test this approach using simulations and empirical data. First, we perform simulations to test our hypothesis that the Mouselab-MDP paradigm yields so much information about how people plan that it becomes possible to accurately infer which planning strategy they used on a single trial and how that strategy differed from the strategies that the participant used on the preceding trial and on the following trial. In follow-up simulations we then assess whether this is also true for the relative contributions of different decision systems. Following these simulation studies, we test whether the inferences of our method are valid measures of planing and learning by applying it to empirical data from studies where planning and learning were experimentally manipulated.

### Simulation studies

To test if our experimental paradigm makes it possible to infer people’s planning strategies on a trial-by-trial basis, we simulated which process-tracing data we would obtain in a Mouselab-MDP experiment depending on which strategies people use and how those strategies change from each trial to the next. We then applied our computational microscope to the simulated process-tracing data to test if that data would be sufficiently informative about the underlying planning strategies that we would be able to infer them correctly. Concretely, we report two sets of simulations suggesting that our method can accurately measure changes in people’s planning strategies and the relative influence of different decision systems, respectively.

#### Is the process-tracing data from the Mouselab-MDP paradigm sufficiently informative about people’s planning strategies?

We simulated a Mouselab-MDP experiment with 31 trials of the 3-step planning task illustrated in Fig. 1 and described in Section 5 for various different sequences of planning strategies. We derived six sets of sequences of planning strategies from five different models of how people might learn how to plan. To generate the first data set, we applied the rational model of strategy selection learning by Lieder and Griffiths (2017); the parameters of this model were fit to the data from 57 participants performing 31 trials of the 3-step planning task illustrated in Fig. 1 (i.e., the control condition of the experiment described in Appendix A.1). We created four additional data sets by modeling the temporal evolution of people’s planning strategies as gradual learning, insight-like learning, a mixture of both gradual and insight-like learning, or a random process that chooses the strategy on each trial independently at random (random model). In all cases, the generation of the strategy sequence and the generation of each click sequence given the sampled strategy involved a considerable amount of randomness that matched or exceeded the variability observed in human data. For a more detailed description of how the data was generated, please see Section A in the Appendix. To avoid bias towards any one of the five models, we used each of them to generate a data set with 500 simulated participants completing 31 trials each. We then combined the resulting five data sets into a single data set from 2500 simulated participants.

We then used our computational microscope to compute the maximum a posteriori estimate of the sequence of strategies for each participant and compared it to the ground truth sequence of strategies. We evaluated the informativeness of our process-tracing paradigm in terms of how accurately the strategies and strategy types could be inferred from the simulated process-tracing data. We found that the process-tracing data made it possible to infer the true strategy for 80 ± 0.01*%* of the trials and to infer the true strategy type for 92 ± 0.00*%* of them. These findings suggest that our experimental paradigm yields so much information that we can hope to be able to infer people’s planning strategies on a trial-by-trial basis. Furthermore, these results suggest that we have implemented our computational method correctly and that the 79 candidate strategies are different enough that it is possible to discern between them. For a detailed description of model-wise strategy and strategy type accuracies, please see Appendix A.3.

#### Validation of measuring the contributions of different decision systems and other factors

**Fig. 11:**
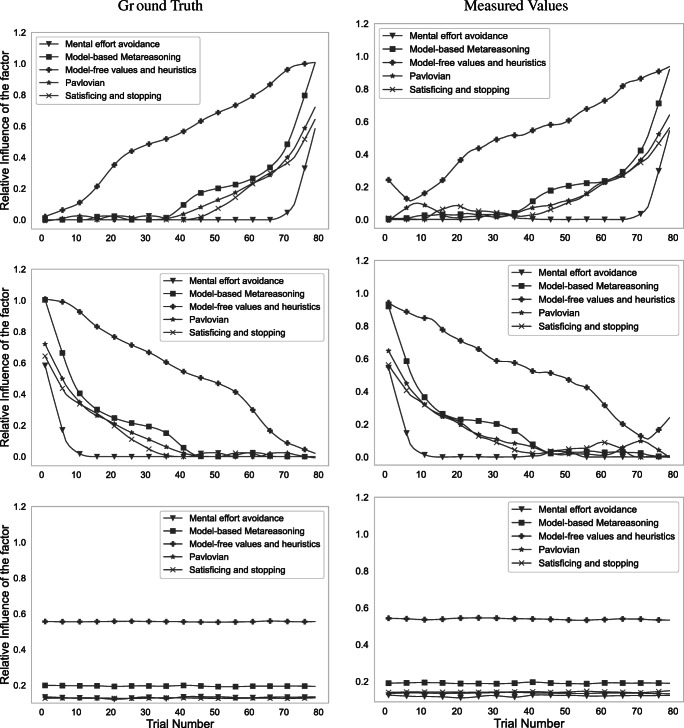
Smoothed plots for comparison of the actual and inferred trends in the relative influence of different decision systems and other factors. The computational microscope was applied to click sequences generated from strategy sequences where the weight of one of the five factors was systematically increasing (top row), decreasing (center row), and constant (bottom row) respectively. Each line is based on a different strategy sequence

We validated our method’s ability to recover the trend in the relative influence of different decision systems and other factors across a series of 79 trials. Each simulation assumed one of three possible trends: increasing influence, decreasing influence, or constant influence. For each factor, for the increasing and decreasing trends, we created a sequence of 79 strategies in which each strategy appears only once and the order of the strategies in the sequence is the sorted order of the contribution of the factor to the corresponding strategy. We then generated a dataset of 500 sequences of click sequences. For the constant case, for each factor, we partitioned the set of strategies into up to 3 groups based on the 33^rd^, 67^th^ and 100^th^ percentiles of the relative influence of the factor across all strategies. We validated our microscope on 500 simulated sequences. To generate a sequence, we randomly selected one of the three groups to generate sequences from and then sampled 79 strategies from that group and arranged them in sequence. Figure 11 shows that our computational microscope recovered the trends in the relative influence of the decision systems and other factors very accurately.

### Validation on empirical data

We also validated our computational microscope on empirical data, that is we tested whether it can detect the effects of experimental manipulations and task structure on people’s planning strategies and metacognitive learning.

#### Detecting the effect of feedback on cognitive plasticity

To verify whether our computational microscope can detect the effect of an experimental manipulation expected to promote cognitive plasticity, namely feedback, we applied it to the Mouselab-MDP process-tracing data from the experiment described in Appendix A where 164 participants solved 30 different 3-step planning problems of the form shown in Fig. 1. Participants in the control condition received no feedback whereas participants in the first experimental condition received feedback on their actions (Action FB) and participants in the second experimental condition received feedback on how they made their decisions (Metacognitive FB). Action FB stated whether the chosen move was sub-optimal and included a delay penalty whose duration was proportional to the difference between the expected returns of the optimal move versus the chose one. In contrast to Action FB, Metacognitive FB pertains to how the decisions are made rather than to the decisions themselves. Metacognitive FB is given after every information gathering operation (click). It has two components that convey the informational value of the planning operation and the planning operation that the optimal strategy would have chosen, respectively.

This metacognitive feedback was designed to be more effective than action feedback at teaching people the optimal planning strategy for the task illustrated in Fig. 1. This strategy (Callaway et al., 2018) starts by searching the potential final destinations for the best possible outcome and terminates planning when it finds one of them.

**Fig. 12:**
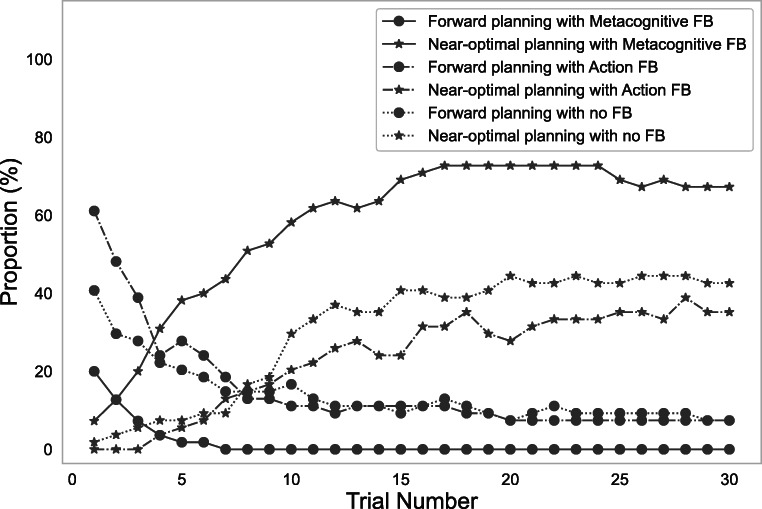
Comparison of frequencies of forward-planning and near-optimal strategies across different types of feedback in the experiment described in Appendix A.1. The green, orange and the blue lines represent the metacognitive feedback, action feedback and the no feedback conditions respectively. The circles represent the forward planning strategies and the stars represent the near-optimal planning strategies

As Fig. 12 shows, the computational microscope correctly detected that feedback boosted metacognitive learning. Concretely, the computational microscope revealed that metacognitive feedback boosted the discovery of the optimal planning strategy (58% vs. 31% in the no feedback condition, *z* = 15.44,*p* < 0.0001, BF > 1000)^2^ and decreased people’s propensity to start planning by considering immediate outcomes, i.e. forward planning (2% vs. 14% in the no feedback condition, *z* = − 13.27,*p* < 0.0001,BF > 1000) whereas action feedback reduced the frequency of the near-optimal planning strategy (24% vs. 31% in the no feedback condition, *z* = − 4.74,*p* < 0.0001,BF > 1000) and did not change the frequency of the forward planning strategies (15% vs. 16% in the no feedback condition, *z* = 1.00,*p* = 0.3193,BF = 0.10 ).

**Fig. 13:**
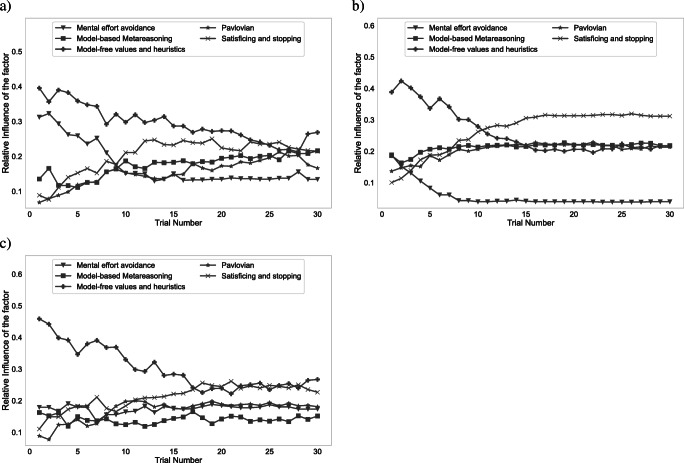
Temporal evolution of the relative influence of different decisions systems and other factors in the control condition without feedback (**a**), the experimental condition with metacognitive feedback (**b**), and the experimental condition with action feedback (**c**), respectively

The computational microscope allows us to gain additional insights into how those changes in people’s strategies come about. Concretely, correcting for multiple comparisons (*α*_*sidak*_ = 0.0034) and applying Wilcoxon-signed rank test, Fig. 13 shows that metacognitive feedback significantly accelerated people’s transition to choosing their planning operations increasingly more based on the model-based metareasoning system (*T* = 248,*p* = 0.0004,BF = 65.31), the Pavlovian system (*T* = 276,*p* = 0.0007,BF = 38.15), and the system for deciding when to stop planning (*T* = 82,*p* < 0.0001,BF = 23568.70). This makes sense because the structure of the environment makes it beneficial to inspect nodes that are most uncertain (a feat accomplished by the metareasoning system), explore nodes that lie on the path to the most valuable nodes (as recommended by the Pavlovian system), and to stop as soon as a very good path has been identified (a feat that accomplished by the system for deciding when to stop). Also, Metacognitive feedback, in general, drove people towards planning more by reducing the amount of mental effort avoidance (*T* = 1.0,*p* = 0.0001,BF = 167.25). Action FB, by contrast,

drove people towards relying more on the Pavlovian system (*T* = 183,*p* = 0.0004,BF = 1236.80), and the decision system for deciding when to stop planning (*T* = 134,*p* = 0.0001,BF = 685.42) and relying less on the model-free values and heuristics (*T* = 229,*p* = 0.0004,BF = 172.56) decision system. In the condition without feedback, people relied increasingly more on the Pavlovian system (*T* = 148,*p* < 0.0001,BF = 1852.39), the system for deciding when to stop planning (*T* = 173,*p* < 0.0002,BF = 647.56) and on the model-based metareasoning system (*T* = 206,*p* = 0.0012,BF = 38.51) but less significantly when compared to the metacognitive feedback condition.

The computational microscope also provides insights into which unique strategy types people go through during learning (learning trajectories) and how this is affected by feedback. Overall, we found that 86% of people’s learning trajectories were unique. However, when we zoom out to the level of strategy types, the computational microscope reveals several common learning trajectories (see Table 3).

**Table 3:** Common trajectories of strategy types by the type of feedback participants received

Condition	Usage %	Strategy type trajectory
No FB		
	7%	FP
	6%	MP → LS → MGS
	6%	MGS
	4%	FP-BFS → MGS
	4%	MS → MGS
MCFB		
	22%	MGS
	16%	MS → MGS
	7%	FGS → MGS
	7%	FP → FGS → MGS
	5%	FP → MGS
Action FB		
	11%	FP
	4%	MP
	4%	FP → MP → FP → MP
	2%	LS → MS → MGS → LS → MS →
		MGS → MS → MGS
	2%	MP → FP → MP → FP → MS → MGS

The strategy types are: FP - Frugal planning, MP - Myopic planning, MGS - Maximizing goal-setting with limited backward planning, LS - Local search, FP-BFS - Forward planning like Best-First search, FGS - Frugal goal-setting strategies, MS - Miscellaneous strategies

We found that the number of strategy types people go through from their initial strategy to the final strategy was lower when participants received metacognitive feedback than when they received action feedback (*t*(107) = − 3.73, *p* = 0.0002,BF = 161.30) or no feedback (*t*(107) = − 2.65,*p* = 0.0046,BF = 8.77). We found no significant difference between the Action FB and the No Feedback conditions (*t*(106) = 1.46,*p* = 0.0737,BF = 0.09)

#### Measuring how people’s planning strategies differ depending on the structure of the environment

Previous work has shown that people adapt their cognitive strategies to the structure of the decision environment (Payne et al., 1993; Callaway et al., 2018; Lieder & Griffiths, 2017; Gigerenzer & Selten, 2002). Here, we verify that our method is able to detect differences in people’s strategies across the four environments described in Section 5. To do so, we applied the computational microscope to the process-tracing data participants generated in the test blocks of the corresponding experiments after they had learned about their respective environment in the training block (see Table 2). Because participants went through a sufficiently large number of training trials, we observed that participants’ planning strategies were stable. As shown in Table 4, the computational microscope revealed that people adapted their planning strategy to the structure of their environment. These differences are systematic in the sense that how people’s strategy choices differ across environments roughly corresponds to how the strategies’ performance differs across those environments. To quantify this, we report the relative performance (*r*^rel^) of the most common strategies relative to the best-performing strategy of each environment. The performance of each strategy (*r*_*i*_) was determined by running 100,000 simulations, and then normalized according to $r^{\text {rel}}_{i}=\frac {r_{i}- \min \limits _{j} r_{j}}{\max \limits _{j} r_{j} - \min \limits _{j} r_{j}}$.

**Table 4:** Summary of the performance of the most frequent strategies across four different environments

Environment	Most common strategies	Relative score (*r*^rel^)	Frequency
Increasing Variance			
(3-steps)			
	Search for the best possible final outcome	1.00	45.1*%*
	Consecutive second maximum	0.93	11.6*%*
	No planning	0.13	10.6*%*
Increasing Variance			
(5-steps)			
	Search for best possible final outcome	1.00	20.8*%*
	No planning	0.28	16.2*%*
	Explore immediate and final outcomes with satisficing on finding a large value	0.97	12.4*%*
	Explore final outcomes and their parents	0.92	11.6*%*
	Explore final outcomes and their parents with satisficing	0.97	6.6*%*
	Explore immediate outcomes on the paths to the best final outcomes	0.99	4.5*%*
Decreasing Variance			
(3-steps)			
	Explore immediate outcomes and final outcomes with satisficing on a positive value	0.97	32.7*%*
	Satisficing Best First Search after exploring all immediate outcomes	0.94	26.7*%*
	No planning	0.00	12.5*%*
	Explore immediate outcomes and final outcomes with satisficing on a positive value	0.96	10.8*%*
	Explore sub-trees of positive immediate outcomes	0.93	5.4*%*
	Explore all immediate outcomes	1.00	5.3*%*
Constant Variance			
(3-steps)			
	Explore all immediate outcomes with satisficing	0.81	17.9*%*
	Satisficing Best First Search after exploring all immediate outcomes	0.91	14.4*%*
	Explore the immediate children of the best immediate outcome	0.81	10.2*%*
	Non-terminating Best First Search	0.74	6.4*%*
	Exploring immediate and final outcomes with positive satisficing	1.00	5.5*%*
	Best First Search after exploring all immediate outcomes	0.75	5.5*%*
	Pruning of nodes with immediate negative rewards and choosing actions with best long-term consequences	0.94	4.8*%*
	Leave out one immediate outcome	0.79	4.4*%*
	Consecutive second maximum	0.85	4.0*%*
	Goal-setting with positive satisficing	0.90	3.0*%*

The performance of each strategy (*r*_*i*_) was determined by running 100,000 simulations, and then normalized according to $r^{\text {rel}}_{i}=\frac {r_{i}- \min \limits _{j} r_{j}}{\max \limits _{j} r_{j} - \min \limits _{j} r_{j}}$. To be included in this table, a strategy had to be used in at least 3*%* of all trials

For both environments with increasing variance, our computational microscope detected that the most common strategy was the near-optimal goal-setting strategy which exploits that the most distant rewards are most variable.

By contrast, people almost never used this strategy in any of the other environments. For the environment with decreasing variance, our computational microscope detected that people primarily use strategies that exploit the structure of this environment by prioritizing its immediate outcomes.

For the environment with constant variance, the computational microscope detected that after inspecting all immediate outcomes the second most frequent strategy performs Best-First Search with Satisficing, which is adaptive in this environment (Callaway et al., 2018), although the most commonly used strategy was not particularly adaptive.

These results show that the computational microscope can reliably reveal how the planning strategies people use differ depending on the structure of the environment. Furthermore, comparing the strategies the computational microscope inferred for the 5-step version of the increasing variance environment that was used as a transfer task to the 3-step version of that environment that was used as a training task suggests that the computational microscope can reveal the transfer of learning across environments.

Equally, the strategy types inferred by our computational microscopes were consistent with previous findings suggesting that people adapt their decision strategies to the structure of the environment (Payne et al., 1993; Callaway et al., 2018; Lieder & Griffiths, 2017; Gigerenzer & Selten, 2002). Table 5 shows the performance and frequency of the inferred strategy types in decreasing order of their frequency for each of the 4 environments. The performance of a strategy type was determined by the weighted average of the performances of the strategies belonging to that strategy type where the weight of a strategy is the relative frequency of the strategy among the strategies belonging to the cluster. As expected, we find that in both increasing variance environments, people primarily rely on strategies that prioritize the potential final outcomes. For the environment with decreasing variance, the computational microscope inferred that most people used the strategy type that is best adapted to this type of environment, namely myopic planning strategies. For the environment with constant variance, the computational microscope inferred that forward planning strategies similar to best first-search was the second most frequently type of planning strategies. The most common strategy type was “Myopic Planning” which includes several strategies that are similar to Best First Search (see Section 5).

**Table 5:** Summary of the performance of the most frequent strategy types for four different environments

Environment	Most common strategy types	Relative score (*r*^rel^)	Frequency
Increasing variance			
(3-steps)			
	Maximizing goal-setting with limited backward planning	1.00	62.9*%*
	Frugal planning strategies	0.00	11.0*%*
	Miscellaneous strategies	0.81	10.2*%*
	Local search strategies	0.92	5.5*%*
	Myopic planning strategies	0.41	4.7*%*
	Frugal goal-setting strategies	0.75	3.8*%*
Increasing variance			
(5-steps)			
	Maximizing goal-setting with limited backward planning	0.86	38.9*%*
	Miscellaneous strategies	0.63	24.6*%*
	Frugal planning strategies	0.00	16.2*%*
	Frugal goal-setting strategies	0.58	6.8*%*
	Immediate rewards on the path to best final outcomes with satisficing	1.00	4.5*%*
	Local search strategies	0.64	4.1*%*
	Myopic planning strategies	0.33	3.2*%*
Decreasing variance			
(3-steps)			
	Myopic planning	1.00	53.4*%*
	Forward planning strategies similar to Best First Search	0.85	33.0*%*
	Frugal planning strategies	0.10	12.6*%*
Constant variance			
(3-steps)^3^			
	Myopic planning	0.76	46.8*%*
	Forward planning strategies similar to Best First Search^4^	1.00	26.8*%*
	Frugal goal-setting strategies	0.65	7.5*%*
	Maximizing goal-setting with limited backward planning	0.75	6.8*%*
	Miscellaneous strategies	0.22	4.6*%*
	Local search strategies	0.40	3.5*%*

^3^ The strategy type with the highest average score was “Frugal planning strategies” (*r*^*rel*^ = 1.00, frequency: 2.1%). Its score is so high because its most frequent strategy is a high-performing strategy similar to Best First Search. This strategy type is not listed because its relative frequency is less than 3%

^4^ The average score of this adaptive strategy type is surprisingly low because it includes strategies that incur a very high planning cost by inspecting all of the information available

The performance of each strategy was determined by running 100,000 simulations. The performance of a strategy type was determined by the weighted average of the performances of the strategies belonging to that strategy type where the weight of a strategy is the relative frequency of the strategy among the strategies of the same type. To be included in this table, a strategy type had to be used in at least 3*%* of all trials

Overall, the results in Tables 4 and 5 illustrate that our computational microscope makes it easy for researchers to describe both the adaptiveness of human planning and its limits.

## Discussion

We have developed a computational process-tracing method that allows us look at how people plan and how their planning strategies change over time. Our method extends the Mouselab paradigm for tracing people’s decision strategies (Payne et al., 1993) in three ways. First, it progresses from one-shot decisions to sequential decision problems. Second, it introduces computational methods for analyzing process tracing data in terms cognitive strategies. Third, we have extend the approach to measuring how people’s planning strategies change over time. Our method is easy to use and freely available. We have successfully evaluated our methods using simulations and human data. The results suggest that our computational microscope can measure cognitive plasticity in terms of the temporal evolution of people’s cognitive strategies and also provide us with valuable information about the trends in changes of strategies, strategy types and also how people change their strategies with changes in environments. We have applied our computational microscope to a number of data sets. The results of these analyses contribute to a more detailed understanding of how people plan and revealed some interesting empirical characteristics of metacognitive learning.

Our method can be used to study many different types of cognitive change across a wide range of different timescales. This makes it suitable for investigating learning, cognitive development, decision-making, individual differences, and psychopathology.

We are optimistic that computational microscopes will become useful tools for investigating the learning mechanisms that enable people to acquire complex cognitive skills and shape the way we think and decide. This will be an important step towards reverse-engineering people’s ability to discover and continuously refine their own algorithms. From a psychological perspective, this line of work might also help us understand why we think the way we do and lead us to rethink our assumptions about what people can and cannot learn. Developmental psychologists could use our method to trace the development of cognitive strategies across the lifespan and elucidate how learning contributes to those developmental changes. Similarly, clinical psychologists and computational psychiatrists could apply it to trace how person’s cognitive strategies changes as they develop and recover from different mental disorders. Importantly, our method can also be used to investigate how cognitive plasticity depends on the learning environment, individual differences, age (Das et al., 2019), time pressure, motivation, and interventions – including feedback, instructions, and reflection prompts. Using our method to measure individual differences in cognitive plasticity might reveal why equivalent experience can have fundamentally different effects on the psychological development of different people. This, in turn, can help us understand why some people are predisposed to develop certain cognitive styles, personalities, and mental disorders. Applications in computational psychiatry might use this approach to understand the development of mental disorders and to create computational assays for detecting whether a person is at risk for developing specific forms of psychopathology long before its symptoms occur.

To facilitate these applications, future work might extend the proposed measurement model to continuous strategy spaces, a wider range of tasks and strategies, and learning at the timescale of individual cognitive operations. In addition, future work will also leverage our computational microscope to elucidate individual differences in cognitive plasticity within and across psychiatric conditions and different age groups. We will also work on making our inferences more precise by learning models of strategies and strategy transitions from human data. To move towards a more naturalistic planning task, future versions of our method could present participants with fully-revealed environments and infer their planning strategies from eye-tracking data. The computational approach could be analogous to the one presented here instead that clicks are replaced by saccades.

The ideas of our approach are not entirely novel. Process-tracing has already been extensively used to study people’s decision strategies (Payne et al., 1993; Schulte-Mecklenbeck et al., 2011; Schulte-Mecklenbeck et al., 2019) and Bayesian inference has been used to infer which decision strategies are include in individual participants’ repertoire (Scheibehenne et al., 2013), when people switch between different decision strategies (Lee & Gluck, 2021), and which strategies people use in economic games Costa-Gomes and Crawford (2006), Crawford (2008), and Costa-Gomes et al., (2001). Our method has several advantages.

What differentiates our approach from the original Mouselab paradigm (Payne et al., 1993) is that it measures how people plan and that we infer people’s strategies from the process-tracing data. On a high level, the Bayesian Toolbox approach by Scheibehenne et al., (2013) also infers people’s strategies. Their approach infers which strategies are included in the person’s repertoire. However, it does not attempt to resolve which strategy was used on which trial. Instead, it makes the simplifying assumption that every decision is influenced by all strategies that are in the person’s toolbox. By contrast, our method makes the different assumption that on each trial each participant draws a single strategy from the toolbox. Based on this assumption, our method infers which individual strategy a participant used on the first trial, which individual strategy they used on the second trial, and so on.

The methods developed by Lee and Gluck (2021) and Lee et al., (2019) are more similar to our method in that they infer which strategy each participant used on each trial of the experiment. The main difference is that these methods were developed for studying multi-cue decision-making whereas our method was developed for studying planning. The method by Lee et al., (2019) has the advantage that it uses process-tracing data, verbal reports, and choices whereas our method exclusively relies on the process-tracing data. While our method and Lee et al., (2019) analyze the data of each participant individually, the method by Lee and Gluck (2021) additionally performs inference at the group level and constrains inferences about individual participants by the characteristics of the group. Furthermore, the method by Lee and Gluck (2021) additionally infers two aspects of the generative model of strategy sequences from the data, namely the probabilities of possible initial strategies and the probabilities of possible strategy transitions. The main advance of our method is that it differentiates between a much larger number of different strategies (79 vs. 4). Furthermore, we examined multiple alternative models of strategy transitions and validated our method on data from multiple different experiments that varied the decision environment and induced systematic learning-induced changes in people’s strategies over time.

Finally, the approaches that have been developed to infer which strategies people use in economic games (Costa-Gomes & Crawford, 2006; Crawford, 2008; Costa-Gomes et al., 2001) assume that each person always uses the same strategy and cannot measure how a person’s strategy changes over time. Furthermore, the strategies these methods measure are specific to strategic social interaction. The strategies people use in tasks such as planning a road trip or project are very different. Therefore, studying them requires a different methodology such as the one we have developed in this work.

In conclusion, the approach introduced in this article complements these existing approaches in useful ways that make it possible to measure people’s planning strategies and how they discover them.

Our methods are not without limitations. First and foremost, the Mouselab-MDP paradigm inherits at least one of the limitations of the Mouselab paradigm that it is based on. Concretely, the Mouselab-MDP paradigm might change how people plan by making information acquisition costlier than it might otherwise be. Previous research comparing Mouselab-based measures of people’s decision processes against equivalent measures based on eye-tracking found that the increased cost of information acquisition in the Mouselab paradigm led people to acquire less information and, to some extent, it also changed the order in which people acquire information (Lohse & Johnson, 1996). We believe that it is likely that similar differences also exist for the Mouselab-MDP paradigm. As Lohse and Johnson (1996) pointed out, such differences are more important for some research questions than for others. Following the logic of their analysis, we believe that there are many important questions about planning and metacognitive learning that are unaffected by such differences. Concretely, our method should be well-suited to characterize the qualitative effects of experimental manipulations on planning and learning as long as it can be expected that the qualitative effects would be the same if the cost of information acquisition was lower. Regardless thereof, we believe that comparing the process-tracing data collected with the Mouselab-MDP paradigm to corresponding process-tracing data based on eye-tracking is an interesting direction for future work.

A perhaps more provocative possibility is that the planning environment that the Mouselab-MDP paradigm seeks to emulate is one in which people cannot simply look up what the outcomes of their actions would be but have to estimate them through effortful mental simulations. In this sense, it is conceivable that the Mouselab-MDP paradigm is closer to the real-world problem that is designed to mimic than an equivalent eye-tracking paradigm would be. This suggest that future work should compare the plans that people arrive at when they have to rely on mental simulations to the plans that they arrive at when those mental simulations are externalized with the Mouselab-MDP paradigm.

One limitation of our computational microscope is that its current implementation requires that the task environment is symmetric and has no circular paths in it. This is because of the features defined in Eq. 1 are computable currently only for such structures. Generalizing the implementation of the computational microscope so that it can be applied to other kinds of environments may be a worthwhile direction for future work.

In summary, our method makes it possible to more directly observe the previously hidden phenomenon of cognitive plasticity in many of its facets – ranging from skill acquisition, learning to think differently, cognitive decline, self-improvement, changes in cognitive dispositions, and the onset, progression, and recovery from psychiatric symptoms and mental disorders. In conclusion, we believe that the method introduced in this paper can be used to advance cognitive science, psychology, and psychiatry in many promising ways.

## Appendix:

### A.1 Experiment (3-step task with increasing variance)

We evaluated our computational microscope on data that was collect in a pilot experiment by Callaway et al., (2022a). The methods and results of that experiment were as follows.

#### Participants

We recruited 164 participants on Amazon Mechanical Turk (average age 35 years, range: 18–72 years; 75 female). Balanced condition assignment and repeat-participant exclusion was performed using Psiturk (Gureckis et al., 2016). None of the participants who finished the experiment were excluded for analysis.

#### Procedure

The experiment comprised instructions, a training block, a test block, and an exit survey. The training block comprised 10 trials, and the test block comprised 20 trials. Each participant was assigned to receive either metacognitive feedback (55 participants), action feedback (55 participants), or no feedback (54 participants) during the training block. The metacognitive feedback used the optimal planning strategy for the environment to provide feedback on participants’ clicks. The action feedback condition provided feedback on the actions (moves) of the participants. The exit survey asked participants about what they had learned, their age, and their gender identity.

#### Materials

Each trial of the experiment presented participants with an instance of the 3-step planning problem described in Fig. 1. The key structure of this problem is that the range of possible rewards is smallest in the first step, larger in the second step, and largest in the third step. To operationalize the cost of planning, we charged participants one virtual dollar per click. To simplify the implementation of metacognitive feedback, we required that all clicks be made before the first move. To eliminate the time cost of engaging in planning compared to speeding through the experiment, participants who spent less than 7 seconds on planning (e.g., only 3 seconds) had to wait for the remaining time after executing their moves (e.g., for 4 seconds). In the test block, participants started with an endowment of 50 virtual dollars and earned a bonus of 1 cents for every $5 they made in the game.

#### Results

In the test block, the average score of participants receiving no feedback was 27.58 points/trial (95% CI: [26.21, 28.95]). Critically, participants receiving metacognitive feedback scored significantly higher (34.86 points/trial; 95% CI: [33.83, 35.89]; *t*(3268) = 8.3268,*p* < 0.001,BF = 5.39 ⋅ 10^13^). By contrast, giving participants conventional feedback on their actions appeared to be ineffective. That is, participants receiving action feedback did not score significantly higher than participants in the no-feedback condition (27.57 points/trial; 95% CI: [26.21, 28.93]; *t*(3268) = 0.0108,*p* = 0.504,BF = 0.039) and performed significantly worse than participants who received metacognitive feedback (*t*(3298) = − 8.3425,*p* < 0.001,BF = 6.13 ⋅ 10^13^).

### A.2 Modeling transitions between planning strategies

The transitions between strategies from one trial to the next define the nature and the type of cognitive plasticity. One possible way in which people might switch from one strategy to the other is based on the similarity of strategies (measured in terms of distance between them). In addition to the similarity of strategies, we have to define how the similarities manifest themselves into the actual transitions. Therefore, we define different measures of similarity and mechanisms of how the distances manifest into strategy transitions.

### A.2.1 Distances

To find out if the frequency of transitions between strategies was dependent on how close the strategies are, we consider 6 metrics of distances between the strategies. Using these distance metrics, we did not find any correlation between the probabilities of transition from one strategy to the other and the distance between them. In this section, we describe each of the three types of metrics and their constituents.

#### 1. Behavioral distances

The behavioral distance between two strategies is measured by the distance between the probability distribution of clicks made by the two strategies. We consider two behavioral distances to quantify the similarity between the strategies.

#### Jensen-Shannon Divergence

6 $$ \begin{array}{@{}rcl@{}} {\Delta}{ (s_1, s_2)} &= &\text{JSD}\left[p(\mathbf{d}|s_1),p(\mathbf{d}|s_2)\right] \\ &=& \text{KL}\left[p(\mathbf{d}|s_1), \frac{p(\mathbf{d}|s_1) + p(\mathbf{d}|s_2)}{2} \right] \\&&+ \text{KL}\left[p(\mathbf{d}|s_2),\frac{p(\mathbf{d}|s_2) + p(\mathbf{d}|s_1)}{2}\right] , \end{array} $$

#### Jeffreys Divergence (Symmetric-KL Divergence)

7 $$ \begin{aligned} {\Delta}{ (s_{1}, s_{2})} &= \text{JD}\left[p(\mathbf{d}|s_{1}),p(\mathbf{d}|s_{2})\right]\\ &= \text{KL}\left[p(\mathbf{d}|s_{1}),p(\mathbf{d}|s_{2})\right] \\&\quad+ \text{KL}\left[p(\mathbf{d}|s_{2}),p(\mathbf{d}|s_{1})\right] \end{aligned} $$

#### 2. Distances in the strategy weight space

This distance metric measures similarity between strategies based on the weight space representation of the strategy. For computing the distance between strategies according to this distance metric, we represent a strategy using its preference for particular features which is quantified using the weights that are applied to the values of the features. Therefore, we quantify similarities between strategies by representing each strategy using a weight vector (*w*) and then measure the similarity of the strategies as the similarity of the weight vectors. We consider two notions of similarity for strategy weight vectors.

#### Manhattan distance in the strategy weight space

8 $$ \begin{array}{@{}rcl@{}} {\Delta}{ (s_1, s_2)} = \left\lVert w_1 - w_2\right\rVert_{1} , \end{array} $$

#### Euclidean distance in the strategy weight space

9 $$ \begin{array}{@{}rcl@{}} {\Delta}{ (s_1, s_2)} = \left\lVert w_1 - w_2\right\rVert_{2} , \end{array} $$

where $\left \lVert x\right \rVert _{p}$ represents the p-norm of the vector *x*, i.e.

10 $$ \begin{array}{@{}rcl@{}} \left\lVert x\right\rVert_{p} = (\sum\limits_{i=1}^{n} |x_i|^{p})^{1/p} \end{array} $$

#### 3. Distances in the decision system weight space

This distance metric measures similarity between strategies based on the contribution of various decision systems to the strategies. To compute the distance between strategies according to this distance metric, we represent a strategy using its preference for one decision system over the other. This preference is quantified using the total preference over all the features of the decision system. Therefore, we measure similarities between strategies by representing each strategy using a weight vector (*w*^*d**s*^) where each weight in the vector quantifies a preference for a decision system and then measure the similarity of the strategies as the similarity of the decision weight vectors. We consider two notions of similarity for strategy decision system weight vectors.

#### Manhattan distance in the decision system weight space

11 $$ \begin{array}{@{}rcl@{}} {\Delta}{ (s_1, s_2)} = \left\lVert w^{\text{ds}}_1 - w^{\text{ds}}_2\right\rVert_{1} , \end{array} $$

#### Euclidean distance in the decision system weight space

12 $$ \begin{array}{@{}rcl@{}} {\Delta}{ (s_1, s_2)} = \left\lVert w^{\text{ds}}_1 - w^{\text{ds}}_2\right\rVert_{2} , \end{array} $$

### A.2.2 Priors

In this section, we define how the distances between strategies manifest into transitions between them. For this purpose, we consider two priors on the strategy transitions that use the distances defined in Appendix A. They are the gradual learning prior and the mixed prior. We describe each of them in turn.

#### Gradual learning prior

The gradual learning prior (*m*_gradual_ in Eq. 13) assumes that strategies change gradually (i.e transitions to strategies that are close-by in terms of the distance metrics when compared to other strategies).

13 $$ \begin{array}{@{}rcl@{}} P(S_{t+1} = s|S_t,m_{\text{gradual}}) = \frac{ \exp(-\frac{1}{\tau} {\Delta}(s,S_t))}{{\sum}_{s^{\prime} \in \mathcal{S}} \exp(-\frac{1}{\tau} {\Delta}(s^{\prime},S_t))}, \end{array} $$

#### Mixed prior

The mixed prior (*m*_mixed_ in Eq. 14) assumes that both insight-like (*m*_*i**n**s**i**g**h**t*_ in Eq. 2) and gradual changes coexist.

14 $$ \begin{array}{@{}rcl@{}} P(S_{t+1}&=&s|S_t,m_{\text{mixed}})\\ &=& p_{\text{gradual}}{}P(S_{t+1}=s|S_t,m_{\text{gradual}}) \\&&+ (1-p_{\text{gradual}})P(S_{t+1}=s|S_t,m_{\text{abrupt}}). \end{array} $$

In Eqs. 13 and 14, *τ* is the temperature parameter which balances how much the distance between the strategies effects the transition probabilities between them. We found that fitting *τ* did not improve our inferences. Therefore, we set its value to 1.

We evaluated all the distance metrics defined in the Section A to verify if the gradual and mixed priors with those distance metrics improved our inferences on the strategies. Model selection revealed that the model without a prior based on the strategy distances (i.e. insight-like transition prior) performed better than the model with the gradual and mixed transition priors.

### A.3 Simulated data

To validate our computational microscope on simulated data, we generated data from 5 models: the random model (that selects one strategy at random in each of the trial), the insight model which generates click sequences according to Eq. 2, the gradual model described in Eq. 13, the mixed model described in Eq. 14 and the RSSL model based on Lieder and Griffiths (2017). The first four models repeat the previous strategy with a probability of 50% and samples it from its model of strategy change otherwise. According to a mixed model, there is a 50% chance that such strategy changes will follow the gradual model (13) and a 50% chance that they will follow the insight model (2). The RSSL model treats the problem of deciding how to plan as a 79-armed bandit with one arm for each strategy. It performs Bayesian inference on the expected return of each strategy and selects strategies via Thompson sampling. It has 79 × 2 = 158 free parameters that specify the prior mean and variance of each strategy’s expected return. These parameters of the model were fit using data from Lieder (2018b). In all cases, the simulation of how the simulated strategies generate click sequences was also probabilistic. Concretely, the click sequences were generated according to the probabilistic soft-max model of the corresponding strategy (1). For each strategy the temperature parameter (*τ*) that determines the amount of randomness in the generation of the click sequences was estimated from human data.

We found that for data generated from the random model, our computational microscope correctly inferred the true strategy for 76 ± 0.007*%* of the trials and correctly predicted the strategy type for 91 ± 0.005*%* of the trials. Similarly for the gradual learning model, our computational microscope could correctly predict the true strategy in 77 ± 0.007*%* of the trials and the correct strategy in 91 ± 0.004*%* of the trials. For data generated from the insight-like learning model, our method correctly predicted the ground-truth strategy in 88 ± 0.005*%* of the trials and the ground-truth strategy type in 96 ± 0.003*%* of the trials. For the data generated from the model which is assumes a combination of gradual learning and abrupt insights, our method correctly inferred the true strategy in 82 ± 0.006*%* of the trials and the true strategy type in 94 ± 0.004*%* of the trials. For data generated from the RSSL model, our computational microscope was able to correctly infer the true strategy in 76 ± 0.007*%* of the trials and the true strategy type in 90 ± 0.005*%* of the trials.

### A.4 Planning strategies

We have considered a total of 79 strategies clustered into 13 types of strategies. The clustering was done by applying Ward’s hierarchical clustering technique to the strategies’ pairwise distances computed by the Symmetric Kullback Leibler Divergence between the probability distributions of clicks induced by the strategies. Since the output of the clustering is a hierarchical partitioning of the set of strategies, we chose the level of hierarchy that gave us the most interpretable clusters. This section describes the strategy types and the strategies that belong to each type. Please note that the strategies are stochastic and the description only corresponds to the actual behavior most of the time.

All of the strategies described below make the best use of the available information to make the final move. That is, the strategies take the path that has the highest expected value.

**Goal-setting with exhaustive backward planning**

These strategies explore all the outcomes. They start by exploring the final outcomes and then plan backwards . They differ in when they initiate backward planning. The model-free values and heuristics decision system and the Pavlovian decision system together contribute at least 75% on average to the strategies in this cluster.

#### Strategy 1: Goal-setting

This strategy starts by exploring the final outcomes in a random order and if a positive final outcome is found, it looks at the outcomes randomly along the path to the start node and this procedure is repeated until all outcomes are explored.

#### Strategy 2: Immediate goal setting

This strategy’s behavior is similar to strategy 1 but differs in the way it explores the outcomes on the path to the start node. Instead of exploring randomly on the path to the start node, it explores them level by level.

#### Strategy 3: Immediate goal setting with preference for siblings

This strategy’s behavior is similar to that of strategy 2 except that after exploring the path until the start node, it explores the sibling of the previously observed final outcome.

#### Strategy 4: Immediate goal setting with preference for siblings and immediate ancestors

This strategy’s behavior is similar to that of strategy 3 but more priority is given to immediate ancestors when there are multiple ancestors.

#### A.4.2 Maximizing Goal-setting with exhaustive backward planning

The only strategy in this category first explores all final outcomes and then plans backwards from them in the order of their value until it has explored all the outcomes. The model-free values and heuristics decision system and the Pavlovian decision system together contribute at least 75% on average to the strategy in this cluster.

##### Strategy 5: Maximizing goal-setting with exhaustive backward planning

This strategy starts by exploring the final outcomes and then plans backwards in the decreasing order of values of the final outcomes. This strategy doesn’t terminate until it has observed all the outcomes.

#### A.4.3 Maximizing goal-setting with limited backward planning

These strategies focus their exploration on potential final outcomes and their termination depends on whether or not a high value has been observed. These strategies do not do backward planning, except for strategy 6, which is an approximation to the near-optimal goal-setting strategy for the increasing variance environment for the three step task with increasing variance defined in Section 5. The strategies differ in when they terminate planning, especially with respect to how much they continue exploring after uncovering sufficiently good information. The model-free values and heuristics decision system, the model-based metareasoning decision system and the Pavlovian decision system in combination contribute at least 75% on average to the strategies in this cluster.

##### Strategy 6: Search for the best possible final outcome

This strategy starts with exploring the final outcomes in a random order and terminates clicking upon finding an outcome with value equal to the maximum observable value of the reward distribution. If such a node is not found, it explores all the final outcomes. If there are multiple final outcomes with the same highest observed value, the strategy might do backward planning along the paths from those outcomes. This strategy approximates the optimal strategy for the three-step task with increasing variance.

##### Strategy 7: Excessive goal-setting

This strategy starts with exploring the final outcomes in a random and explores one extra outcome after exploring an outcome that with value equal to the maximum observable value of the reward distribution. If such a node is not found, it explores all the final outcomes and terminates.

##### Strategy 8: Leave out one final outcome

This strategy randomly explores all final outcomes except for one randomly- selected final outcome.

##### Strategy 9: Extra planning after exploring the second best value

This strategy starts by exploring the final outcomes and terminates after exploring one extra final outcome after having found an outcome whose value is greater than the second largest observable value of the reward distribution.

##### Strategy 10: Explore as many final outcomes as there are initial outcomes

This strategy explores as many final outcomes as there are immediate outcomes in the task structure.

##### Strategy 11: One outcome per sub-tree of the start node

This strategy explores one random outcome from each sub-tree of the start node.

##### Strategy 12: Consecutive second maximum

This strategy starts by exploring the final outcomes in a random order and terminates after exploring two outcomes consecutively whose values are greater than the second largest value of the reward distribution.

##### Strategy 13: Explore two extra outcomes after exploring a positive outcome

This strategy starts with exploring final outcomes in a random order and terminates after exploring two extra final outcomes after exploring a positive final outcome.

##### Strategy 14: Immediate outcomes after final outcomes with satisficing

This strategy explores all the final outcomes first and then explores all the immediate outcomes. While exploring the final outcomes, if it finds an outcome whose value is equal to the maximum observable value of the reward distribution, it terminates.

##### Strategy 15: Explore parents of largest final outcomes

This strategy explores all the final outcomes and then explores the parents of the final outcomes with the largest value among the explored outcomes.

#### A.4.4 Frugal goal-setting strategies

These strategies focus their exploration on potential final outcomes but explore less overall. They differ in the way they terminate planning. The model-free values and heuristics decision system, the model-based metareasoning decision system and the Pavlovian decision system in combination contribute at least 75% on average to the strategies in this cluster.

##### Strategy 16: Goal-setting with backward planning

This strategy starts by exploring the final outcomes. It explores the final outcomes until a final outcome with a value equal to the maximum observable value of the reward distribution is explored, plans backwards to the corresponding immediate outcome and then terminates. If such an outcome is not found, it terminates after exploring all final outcomes.

##### Strategy 17: Goal-setting with positive satisficing

This strategy starts with exploring final outcomes and terminates after exploring an outcome whose value is positive. If such an outcome is not found, it explores all the final outcomes and then terminates.

##### Strategy 18: One final outcome

This strategy explores one random final outcome and terminates.

##### Strategy 19: Goal setting with forward planning:

This strategy starts by exploring the final outcomes and after finding an outcome with value equal to the maximum observable value of the reward distribution, it explores the path from the corresponding immediate outcome to that final outcome and then terminates.

##### Strategy 20: Explore one sub-tree

This strategy explores all the outcomes of one random sub-tree of the start node.

##### Strategy 21: Explore parent of the best final outcome

This strategy explores all the final outcomes until it finds a final outcomes whose value is equal to the maximum observable value of the reward distribution and then explores the parent of that outcome.

##### Strategy 22: Explore one path

This strategy explores one random path from an immediate outcome to a final outcome and then terminates.

##### Strategy 23: Two final outcomes

This strategy explores two randomly chosen final outcomes and then terminates.

##### Strategy 24: Explore the parent of a positive final outcome

This strategy starts by exploring the final outcomes in a random order and upon finding a final outcome with a positive value, it explores the parent of that outcome and terminates. If no final outcome with a positive value is found, it explores all final outcomes and then terminates.

##### Strategy 25: Explore all final outcomes of a randomly chosen sub-tree and the parent of a randomly chosen observed final outcome

This strategy explores all the final outcomes of a randomly chosen sub-tree of the start node and then explores the parent of a randomly chosen final outcome from the set of observed final outcomes.

#### A.4.5 Strategy that explores immediate outcomes on the paths to the best final outcomes

The only strategy in this category explores all the final outcomes and then explores the immediate outcomes of the best among them. The model-free values and heuristics decision system and the satisficing and stopping decision system together contribute at least 75% on average to the strategies in this cluster.

##### Strategy 26: Explore immediate outcomes on the paths to the best final outcomes

This strategy starts by exploring all the final outcomes and then explores the immediate outcomes of paths that lead to the best final outcomes.

#### A.4.6 Strategy that explores immediate rewards on the paths to the best final outcomes with satisficing

The only strategy in this cluster behaves similarly to the strategy in the previous category but differs in the fact that it stops exploring the final outcomes after finding an outcome whose value is equal to the maximum observable value of the reward distribution and then explores the immediate outcome of that node. The model-free values and heuristics decision system, the model-based metareasoning decision system and the Pavlovian decision system in combination contribute at least 75% on average to the strategy in this cluster.

##### Strategy 27: Explore immediate outcomes on the paths to the best final outcomes with satisficing

This strategy explores all the final outcomes randomly until it finds a final outcome whose value is equal to the maximum observable value of the reward distribution and then explores the corresponding immediate outcome of that final outcome.

#### A.4.7 Forward planning strategies similar to Breadth First Search

These strategies perform planning similar to Breadth First Search, i.e., they first inspect all outcomes at the first level, before inspecting all outcomes at the second level, and so on. These strategies differ in the order in which outcomes at the same level are explored. The strategy 30 in this category is a satisficing version of breadth-first search which terminates upon finding a high value. The model-based metareasoning decision system alone contributes more than 75% on average to the strategies in this cluster.

##### Strategy 28: Randomized Breadth First Search

This strategy explores outcomes level by level, that is, it randomly explores the outcomes that are one step away, then randomly exploring the outcomes that are two steps away and so on until all nodes are clicked.

##### Strategy 29: Breadth First Search

This strategy behaves similar to strategy 5 except that sibling outcomes are observed consecutively.

##### Strategy 30: Satisficing Breadth First Search

This strategy explores outcome in the breadth-first search order and terminates upon finding the maximum value of the reward distribution.

#### A.4.8 Middle-out planning

The only strategy in this category explores the center outcomes first, then inspects immediate outcomes and finally inspects final outcomes. The model-free values and heuristics decision system and the model-based metareasoning decision system in combination contribute at least 75% on average to the strategy in this cluster.

##### Strategy 31: Middle-out planning

This strategy explores the center nodes first, then explores the immediate outcomes and then the final outcomes.

#### A.4.9 Forward planning strategies similar to Best First Search

These strategies are similar to the Best First Search planning strategy. They differ in how they start and how they terminate. The model-free values and heuristics decision system and the Pavlovian decision system together contribute at least 75% on average to the strategies in this cluster.

##### Strategy 32: Non-terminating Best First Search

This strategy starts by exploring the immediate outcomes and explores an unobserved child of the observed outcome with the highest value. If no outcome is observed, it chooses the immediate outcome randomly.

##### Strategy 33: Best First search after exploring all immediate outcomes

This strategy explores all immediate outcomes first and then follows the best-first strategy (Strategy 32) to explore outcomes further.

##### Strategy 34: Satisficing Best First Search after exploring all immediate outcomes

This strategy is similar to strategy 33 but it stops exploring when an outcome with value equal to the maximum observable value of the rewards distribution is observed.

##### Strategy 35: Explore immediate outcomes and then sub-trees

This strategy explores all immediate outcomes in a random order and then explores all the outcomes of their sub-trees, exploring each sub-tree in a random order.

##### Strategy 36: Explore sub-trees of positive immediate outcomes

This strategy explores all the immediate outcomes and then explores complete sub-trees of the immediate outcomes with a positive value in a random order.

#### A.4.10 Local search strategies

These strategies focus on information about the sub-trees and next/previous steps along the paths that have received the most consideration so far. These strategies differ in whether they prioritize sub-trees or paths and whether earlier versus later outcomes have already been observed. The model-free values and heuristics decision system and the satisficing and stopping decision system together contribute at least 75% on average to the strategies in this cluster.

##### Strategy 37: Progressive Deepening

This strategy is similar to Depth First Search (i.e., it starts with exploring the nodes level by level, first observing the node and then its children) but instead of choosing a sibling of a final outcome, it chooses to explore a path starting from the immediate outcome again.

##### Strategy 38: Priority to explored ancestors

This strategy randomly selects the first outcome to explore. Based on the outcomes explored, this strategy prioritizes exploring outcomes that have a larger number of observed ancestors than the number of explored successors.

##### Strategy 39: Priority to explored successors

This strategy randomly selects the first outcome to explore. Based on the outcomes explored, this strategy prioritizes exploring outcomes that have larger number of observed successors than the number of explored ancestors.

##### Strategy 40: Priority to explored immediate ancestors

This strategy is similar in behavior to strategy 38 but an outcome is given higher priority if it has higher number of immediate ancestors than immediate successors. If the number of immediate ancestors and ancestors and successors is equal, then it prioritizes total number of ancestors over total number of successors.

##### Strategy 41: Priority to explored immediate successors

This strategy is similar in behavior to strategy 39 but an outcome is given higher priority if it has higher number of immediate successors than immediate ancestors. If the number of immediate ancestors and ancestors and successors is equal, then it prioritizes total number of ancestors over total number of successors.

##### Strategy 42: Satisficing Depth First Search

This strategy’s behavior is similar to that of Depth First Search but it terminates upon finding an outcome with value equal to the maximum observable value of the reward distribution.

##### Strategy 43: Leave out one sub-tree

This strategy explores all sub-trees of the start node except one in a random order, exploring all the outcomes of a sub-tree in a random order and then moving on to the next, while exploring the outcomes in each sub-tree also in a random order.

##### Strategy 44: Explore all sub-trees

This strategy explores all sub-trees of the start node in a random order, exploring all the outcomes of a sub-tree in a random order and then moving on to the next, while exploring the outcomes in each sub-tree also in a random order.

##### Strategy 45: Explore all sub-trees with satisficing

This strategy’s behavior is similar to that of strategy 44 but it terminates planning upon finding an outcome that has value equal to the maximum observable value of the reward distribution.

##### Strategy 46: One complete sub-tree and final outcomes of other sub-trees

This strategy explores one random sub-tree of the start node and then explores final outcomes of the other sub-trees.

##### Strategy 47: Two complete sub-trees and final outcomes of the last sub-tree

This strategy explores all the outcomes of two sub-trees randomly, exploring one after the other and then explores the final outcomes of the other sub-trees.

##### Strategy 48: Explore all sub-trees until the maximum value of the reward distribution is found and then explore the center outcome of an unobserved immediate outcome

This strategy explores the outcomes of sub-trees of the start node in a random order and if it finds a final outcome with a value equal to the maximum observable value of the reward distribution, it explores the center outcome on the path from the final outcome to the corresponding immediate outcome and then terminates.

#### A.4.11 Frugal planning strategies

These strategies explore very little or not at all. They differ in which outcomes they inspect and when they terminate. The model-free values and heuristics decision system and the mental effort avoidance decision system together contribute at least 75% on average to the strategies in this cluster.

##### Strategy 49: Myopic Impulsive

This strategy explores one randomly chosen immediate outcome and then terminates.

##### Strategy 50: No planning

This strategy does not plan at all (i.e does not explore any outcomes).

##### Strategy 51: Explore immediate outcomes and final outcomes with satisficing on a positive value

This strategy explores all the immediate outcomes until an outcome with a positive value is found and then explores the final outcomes reachable from that immediate outcome and explores them until a final outcome with positive value is found. If it doesn’t find an immediate outcome with a positive value, it terminates.

##### Strategy 52: Explore one center outcome

This strategy chooses a random path and then explores the center outcome of that path.

#### A.4.12 Myopic planning strategies

These strategies start with exploring immediate outcomes and then explore the sub-trees of the best immediate outcomes. They differ in how many immediate outcomes they explore, which nodes in the sub-tree they explore and when they terminate. The model-free values and heuristics decision system, the model-based metareasoning decision system and the Pavlovian decision system in combination contribute at least 75% on average to the strategies in this cluster.

##### Strategy 53: Explore all immediate outcomes with satisficing

This strategy starts with exploring the immediate outcomes and terminates upon finding an immediate outcome which has a positive value. If an immediate outcome with a positive value is not found, it terminates after exploring all immediate outcomes.

##### Strategy 54: Explore all immediate outcomes

This strategy explores all immediate outcomes and then terminates.

##### Strategy 55: Pruning of nodes with immediate negative rewards and choosing actions with best long-term consequences

This strategy first explores all the immediate outcomes and then for immediate outcomes with a positive value, it explores the corresponding final outcomes such that sibling outcomes are explored consecutively.

##### Strategy 56: Explore positive immediate outcomes and final outcomes with satisficing

This strategy’s behavior is similar to strategy 55 but instead of observing all the final outcomes, the strategy terminates after finding the outcome with a value equal to the maximum observable value of the reward distribution.

##### Strategy 57: Leave out one immediate outcome

This strategy explores all immediate outcomes except for one. The left-out immediate outcome is randomly selected.

##### Strategy 58: Explore immediate and final outcomes with satisficing on finding a large value

This strategy starts by exploring immediate outcomes. It first explores an immediate outcome and then explores the final outcomes of the corresponding immediate outcome. If it finds an outcome with a value that is equal the maximum observable value of the reward distribution while exploring the final outcomes, it terminates.

##### Strategy 59: Explore immediate and final outcomes with positive satisficing

This strategy starts by exploring immediate outcomes. It first explores an immediate outcome and if it has a positive value, it explores the final outcomes of the corresponding immediate outcome and this pattern is repeated. If it finds an outcome with a positive value while exploring the final outcomes, it terminates.

##### Strategy 60: Explore the sub-tree which contains largest final outcome

This strategy explores all the final outcomes in a random order and then explores all the outcomes of sub-trees which contain the final outcome with the largest value.

##### Strategy 61: Explore the immediate children of the best immediate outcome

This strategy explores all the immediate outcomes and then explores a single child of the immediate outcome with the largest value.

##### Strategy 62: Explore final outcomes with preference for nodes in the same sub-tree of the root

This strategy explores the final outcomes, exploring all the final outcomes of one sub-tree before moving on to the next, and terminates when it finds an outcome whose value is equal to the maximum observed value of the reward distribution.

#### A.4.13 Other miscellaneous strategies

These strategies do not fit the definition of any of the above categories and appear to have little in common. The model-free values and heuristics decision system, the model-based metareasoning decision system and the satisficing and stopping decision system in combination contribute at least 75% on average to the strategies in this cluster.

##### Strategy 63: Inverse randomized Breadth First Search

This strategy explores all the outcomes level by level, exploring the farthest ones and moving on to the closer ones, that is, exploring the outcomes that are three steps away (the farthest nodes), then exploring outcomes that are two steps away and so on until all outcomes are explored.

##### Strategy 64: Explore immediate outcomes of final outcomes

This strategy observes all the final outcomes first and then, in the decreasing order of the outcomes values,observes the immediate outcomes.

##### Strategy 65: A version of goal-setting that chooses between equally-good goals based on the immediate reward

This strategy explores all the final outcomes first and then compares paths of the final outcomes with the largest values level by level from the final outcomes to the immediate outcomes.

##### Strategy 66: Goal-setting with comparison of equivalent goals

This strategy’s behavior is similar to that of strategy 65 but the outcomes on the paths to the immediate outcome are explored in a random order.

##### Strategy 67: Best Final Outcome

This strategy explores all final outcomes in a random order and then terminates.

##### Strategy 68: Random planning

This strategy explores outcomes in such a way that there is an equal probability of exploring a given outcome and terminating planning.

##### Strategy 69: Explore immediate outcomes of second best nodes

This strategy first explores all the final outcomes and then explores immediate outcomes of the final outcomes with second-largest value.

##### Strategy 70: Explore immediate outcomes and final outcomes

This strategy first explores all the immediate outcomes in a random order and then explores all the final outcomes in a random order.

##### Strategy 71: Explore immediate outcomes and final outcomes with termination

This strategy first explores all immediate outcomes in a random order and then explores the final outcomes outcomes in a random order. While exploring the final outcomes, if an outcome whose value is equal to the maximum observed value of the reward distribution is found, it terminates.

##### Strategy 72: All immediate outcomes after all final outcomes

This strategy explores all the final outcomes first and then explores all the immediate outcomes.

##### Strategy 73: Explore immmediate, final and center outcomes

This strategy first explores all the immediate outcomes, then explores all the final outcomes and then explores all the center outcomes.

##### Strategy 74: Explore all center outcomes

This strategy explores all center outcomes and then terminates.

##### Strategy 75: Explore the path to the final outcome with largest value and satisficing

This strategy explores final outcomes until it finds a final outcome whose value is equal to the maximum observable value of the reward distribution and then explores the outcomes on the path from that outcome to the corresponding immediate outcome in a random order. After exploring the immediate outcome, it terminates.

##### Strategy 76: Explore center outcomes and then final outcomes

This strategy explores all the center outcomes in a random order and then explores all the final outcomes in a random order.

##### Strategy 77: Explore center outcomes and one of their children

This strategy explores one center outcome and then explores one of its randomly-chosen children and then repeats this process until all center nodes are explored.

##### Strategy 78: Explore final outcomes and their parents

This strategy first explores final outcomes of a sub-tree of the start node and then explores the parent of the explored final outcomes and then repeats this process for all of the sub-trees.

##### Strategy 79: Explore final outcomes and their parents with satisficing

This strategy’s behavior is similar to that of strategy 78 but it terminates when it finds a final outcome whose value is equal to the maximum observable value of the reward distribution.

#### A.4.14 Identifiability and confidence

To estimate how accurately and how confidently individual strategies can be distinguished based on a single click sequence, we compared how probable the click sequence generated by one strategy is under other strategies compared to its likelihood under the true strategy. Our procedure was as follows: For each strategy described in Section A, we generated 1000 click sequences by applying the strategy to 1000 different instances of a given environment. Then, for each click sequence **d** (one simulation), we evaluated whether our method correctly inferred the strategy that generated it and computed the likelihoods with which each of the 79 strategies would generate that click sequence (i.e., *P*(**d**|*s*_1_), *P*(**d**|*s*_2_), ⋯, *P*(**d**|*s*_79_)). We then compute the relative likelihood of the click sequence under each strategy by dividing the likelihood of the click sequence under that strategy by the maximum likelihood for that click sequence under all the strategies (i.e., $\frac {P(\mathbf {d} | s_{1})}{\max \limits _{i} P(\mathbf {d} | s_{i})}, \frac {P(\mathbf {d} | s_{2})}{\max \limits _{i} P(\mathbf {d} | s_{i})}$, etc.). To get a representative statistic of how likely click sequences generated by one strategy are under other strategies, we compute the average of the relative likelihoods obtained for the 1000 simulations (i.e., $\rho _{j,k}=\frac {1}{1000}\cdot {\sum }_{t=1}^{1000} \frac {P(\mathbf {d}_{j,t} | s_{k})}{\max \limits _{i} P(\mathbf {d}_{j,t} | s_{i})}$ where *j* is the strategy that generated the click sequence and *k* is the strategy whose average relative likelihood is being evaluated). In addition, we estimated how confident our method is in each of its inferences by computing the ratio of likelihood of the inferred strategy over the likelihood of the second most likely strategy (LR_1,2_). We ran this procedure for two environments: the 3-step increasing variance environment and the 5-step transfer task.

The results of this evaluation for the three step environment and the five step environment are summarized in Tables 6 and 7, respectively. The first column reports the strategy that generated the data. The second column reports our method’s typical confidence in its inferences in terms of the median of the 1000 LR_1,2_ ratios. The third column reports how often the strategy that our method inferred was identical to the strategy that had generated the data. The fourth column lists other strategies that our method considers to be possible alternative explanations because they are at least 66% as likely as the true strategy at least half of the time. The last five columns show the top 5 average relative likelihood ratios for click sequences generated from a given strategy for the 3-step environment with increasing variance and the transfer task respectively. That is, for the strategy in row *j*, the entries in the columns labelled “1”, “2”, ⋯, “5” are the values of $\rho _{j,k_{1}}, \rho _{j,k_{2}}, \cdots , \rho _{j,k_{5}}$ for the strategy with the highest, second highest, ⋯, tenth highest average likelihood ratio for the click sequences generated by strategy *j*, respectively. In each row, the average likelihood ratio of the true strategy is highlighted in bold.

**Table 6:** Summary of the likelihood of click sequences on the 3-step increasing variance environment

Strategy	LR_1,2_	True proportion	Similar strategies	1	2	3	4	5
1	1.0 ⋅ 10^6^	0.866	None	**0.885**	0.105	0.039	0.039	0.026
2	7.7	0.822	None	**0.866**	0.311	0.311	0.208	0.109
3	1.0	0.985	4	**0.988**	0.988	0.303	0.106	0.038
4	1.0	0.993	3	**0.991**	0.991	0.303	0.092	0.038
5	2.3	0.932	None	**0.955**	0.224	0.213	0.099	0.099
6	1.0	0.751	14,17,18,79	**0.829**	0.729	0.471	0.294	0.256
7	1.1	0.643	67	**0.723**	0.269	0.265	0.244	0.185
8	3.4	0.976	None	**0.982**	0.281	0.133	0.087	0.073
9	1.8	0.652	None	**0.754**	0.447	0.141	0.126	0.117
10	2.5	0.598	None	**0.755**	0.372	0.136	0.101	0.093
11	2.5	1.0	None	**1.000**	0.400	0.068	0.059	0.059
12	1.8	0.773	None	**0.835**	0.451	0.108	0.101	0.096
13	1.6	0.149	None	**0.410**	0.291	0.272	0.229	0.175
14	1.0	0.887	6,17,18,79	**0.920**	0.735	0.474	0.284	0.250
15	7.2 ⋅ 10^4^	1.0	None	**1.000**	0.070	0.046	0.013	0.000
16	2860.0	0.989	None	**0.991**	0.229	0.176	0.176	0.176
17	1.0	0.914	6,14,18,23,79	**0.940**	0.481	0.470	0.463	0.265
18	26	1.0	None	**1.000**	0.485	0.222	0.222	0.222
19	2860.0	0.991	None	**0.992**	0.177	0.177	0.177	0.177
20	4.3	0.949	None	**0.958**	0.176	0.150	0.101	0.072
21	1.0	0.933	24	**0.946**	0.451	0.174	0.174	0.174
22	2860.0	1.0	None	**1.000**	0.241	0.018	0.000	0.000
23	2.4	0.928	None	**0.947**	0.229	0.170	0.158	0.158
24	5.0	0.953	None	**0.962**	0.456	0.046	0.014	0.010
25	2860.0	1.0	None	**1.000**	0.050	0.035	0.000	0.000
26	7.2 ⋅ 10^4^	1.0	None	**1.000**	0.174	0.103	0.059	0.025
27	572.0	0.989	None	**0.991**	0.176	0.176	0.176	0.176
28	2.4 ⋅ 10^5^	0.935	None	**0.939**	0.236	0.062	0.003	0.001
29	15.0	0.976	None	**0.982**	0.065	0.028	0.016	0.009
30	2.4 ⋅ 10^5^	0.968	None	**0.971**	0.222	0.020	0.016	0.001
31	2.4 ⋅ 10^5^	1.0	None	**1.000**	0.000	0.000	0.000	0.000
32	3.2 ⋅ 10^7^	0.999	None	**0.998**	0.247	0.067	0.011	0.007
33	18.0	0.997	None	**0.995**	0.241	0.238	0.028	0.004
34	1.0 ⋅ 10^5^	1.0	None	**0.996**	0.239	0.057	0.035	0.013
35	8.0 ⋅ 10^5^	0.999	60	**0.999**	0.112	0.001	0.001	0.000
36	1.0	0.986	None	**0.988**	0.373	0.132	0.132	0.132
37	9.3	0.994	None	**0.994**	0.015	0.015	0.005	0.001
38	1.2	0.814	None	**0.863**	0.201	0.136	0.055	0.017
39	1.4	0.825	None	**0.870**	0.187	0.139	0.053	0.023
40	6.0	0.997	None	**0.995**	0.196	0.012	0.010	0.008
41	6.0	0.985	None	**0.984**	0.207	0.015	0.015	0.012
42	50.8	1.0	None	**1.000**	0.226	0.032	0.015	0.015
43	3.1	0.627	None	**0.720**	0.220	0.161	0.161	0.146
44	2.9	0.801	None	**0.836**	0.180	0.165	0.150	0.146
45	2.6	0.677	None	**0.765**	0.164	0.164	0.157	0.152
46	548.7	0.729	None	**0.789**	0.168	0.156	0.066	0.052
47	245.0	0.907	None	**0.934**	0.163	0.031	0.030	0.023
48	5.7	0.693	None	**0.777**	0.227	0.166	0.111	0.083
49	1.0	1.0	53	**1.000**	0.502	0.019	0.000	0.000
50	13.0	1.0	None	**1.000**	0.077	0.000	0.000	0.000
51	2860.0	1.0	None	**1.000**	0.242	0.219	0.132	0.132
52	52.0	1.0	None	**1.000**	0.019	0.000	0.000	0.000
53	1.0	1.0	49, 54, 57	**1.000**	0.488	0.262	0.250	0.132
54	2860.0	1.0	None	**1.000**	0.254	0.132	0.132	0.132
55	1.0	1.0	51, 56	**1.000**	0.614	0.255	0.132	0.132
56	1.0	1.0	51, 55	**1.000**	0.610	0.226	0.143	0.132
57	286.0	1.0	None	**1.000**	0.239	0.003	0.000	0.000
58	2.4	0.899	None	**0.912**	0.425	0.103	0.056	0.044
59	4.0	0.898	None	**0.931**	0.433	0.066	0.057	0.022
60	1.2 ⋅ 10^5^	0.964	None	**0.975**	0.374	0.039	0.000	0.000
61	2.6 ⋅ 10^4^	1.0	None	**1.000**	0.000	0.000	0.000	0.000
62	2.9	1.0	None	**1.000**	0.087	0.042	0.036	0.014
63	2.4 ⋅ 10^5^	0.994	None	**0.990**	0.093	0.004	0.002	0.000
64	1.5 ⋅ 10^4^	0.963	None	**0.984**	0.218	0.018	0.018	0.018
65	2.0	0.989	None	**0.987**	0.302	0.088	0.073	0.073
66	275.3	0.651	7, 69	**0.795**	0.328	0.032	0.032	0.019
67	1.0	0.965	None	**0.970**	0.806	0.274	0.238	0.174
68	6.8 ⋅ 10^29^	0.786	None	**0.795**	0.084	0.037	0.029	0.026
69	1.0	0.965	67	**0.973**	0.799	0.174	0.160	0.091
70	2.4 ⋅ 10^5^	0.985	None	**0.986**	0.255	0.020	0.007	0.003
71	20.6	0.859	None	**0.912**	0.244	0.143	0.102	0.048
72	2.4 ⋅ 10^5^	1.0	None	**1.000**	0.185	0.103	0.023	0.000
73	2.4 ⋅ 10^5^	1.0	None	**1.000**	0.000	0.000	0.000	0.000
74	2860.0	1.0	None	**1.000**	0.000	0.000	0.000	0.000
75	3872.8	0.984	None	**0.988**	0.237	0.177	0.177	0.177
76	2.4 ⋅ 10^5^	1.0	None	**1.000**	0.000	0.000	0.000	0.000
77	1.8 ⋅ 10^5^	1.0	None	**1.000**	0.000	0.000	0.000	0.000
78	2.2 ⋅ 10^7^	1.0	None	**1.000**	0.185	0.000	0.000	0.000
79	2.9	1.0	None	**1.000**	0.283	0.283	0.271	0.245

This table summarizes likelihood of click sequences generated using a given strategy and the average relative likelihoods of the generated click sequences under the top 5 strategies on the 3-step increasing variance environment. Each row describes the results for a given strategy. The column LR describes the median ratio of the likelihoods of the first-best strategy to the second-best strategy computed for each simulation separately. The columns 1-5 describe the decreasing order of the average relative likelihoods under the top 5 strategies The numbers in bold represent the average likelihood ratios of the strategy that the simulations were generated from. The column “True proportion” describes the proportion of click sequences for which the true strategy was the most likely strategy. The column “Similar strategies” describes the strategies that the strategy was confused with on more than 20% of the click sequences

**Table 7:** Summary of the likelihood of click sequences on the 5-step transfer task

Strategy	LR_1,2_	True proportion	Similar strategies	1	2	3	4	5
1	4.0 ⋅ 10^31^	1.0	None	**1.000**	0.000	0.000	0.000	0.000
2	4.6 ⋅ 10^22^	1.0	None	**1.000**	0.000	0.000	0.000	0.000
3	9.8 ⋅ 10^30^	0.994	None	**0.994**	0.019	0.000	0.000	0.000
4	1.2 ⋅ 10^29^	1.0	None	**1.000**	0.017	0.000	0.000	0.000
5	1.1 ⋅ 10^22^	1.0	None	**1.000**	0.000	0.000	0.000	0.000
6	1.0	0.987	14,17,18,19,27	**0.989**	0.848	0.621	0.530	0.333
7	6.7 ⋅ 10^6^	0.982	None	**0.982**	0.088	0.085	0.064	0.064
8	1.7 ⋅ 10^13^	1.0	None	**1.000**	0.000	0.000	0.000	0.000
9	1.7 ⋅ 10^8^	0.996	None	**0.997**	0.002	0.002	0.001	0.000
10	249.3	0.999	None	**0.999**	0.047	0.047	0.043	0.042
11	1.9 ⋅ 10^4^	1.0	None	**1.000**	0.003	0.003	0.002	0.002
12	91.0	0.997	None	**0.997**	0.048	0.011	0.005	0.000
13	1.9 ⋅ 10^4^	0.936	None	**0.939**	0.024	0.014	0.013	0.012
14	1.0	0.982	6,17,19,27	**0.986**	0.664	0.646	0.509	0.225
15	1.0	0.991	66,67,69,75	**0.994**	0.523	0.521	0.512	0.363
16	1.1 ⋅ 10^15^	0.996	None	**0.996**	0.007	0.000	0.000	0.000
17	1.0	0.993	6,14,18,19,23,24	**0.994**	0.801	0.727	0.320	0.295
18	1.0	0.992	6,17,24	**0.994**	0.728	0.613	0.209	0.196
19	1.0	0.995	6,14,17,27	**0.995**	0.857	0.551	0.500	0.269
20	753.6	0.999	None	**0.998**	0.046	0.005	0.001	0.000
21	9.6 ⋅ 10^10^	1.0	None	**1.000**	0.003	0.000	0.000	0.000
22	1.7 ⋅ 10^13^	1.0	None	**1.000**	0.000	0.000	0.000	0.000
23	105.1	0.99	None	**0.991**	0.276	0.222	0.201	0.164
24	1.0	0.985	17,18,23	**0.987**	0.807	0.585	0.217	0.129
25	2.0 ⋅ 10^10^	1.0	None	**1.000**	0.001	0.000	0.000	0.000
26	1.6 ⋅ 10^10^	1.0	None	**1.000**	0.106	0.093	0.013	0.004
27	1.0	0.994	6,14,19	**0.995**	0.662	0.531	0.531	0.155
28	9.4 ⋅ 10^15^	1.0	None	**1.000**	0.000	0.000	0.000	0.000
29	5.6 ⋅ 10^10^	1.0	None	**1.000**	0.000	0.000	0.000	0.000
30	6.3 ⋅ 10^6^	1.0	None	**1.000**	0.000	0.000	0.000	0.000
31	3.0 ⋅ 10^17^	1.0	None	**1.000**	0.000	0.000	0.000	0.000
32	1.1 ⋅ 10^31^	1.0	None	**1.000**	0.000	0.000	0.000	0.000
33	1.4	0.979	None	**0.936**	0.578	0.000	0.000	0.000
34	1.4	0.949	None	**0.958**	0.575	0.000	0.000	0.000
35	9.6 ⋅ 10^28^	1.0	None	**1.000**	0.000	0.000	0.000	0.000
36	1.0	1.0	54	**1.000**	1.000	0.128	0.128	0.000
37	4.1 ⋅ 10^22^	1.0	None	**1.000**	0.000	0.000	0.000	0.000
38	1.8 ⋅ 10^19^	1.0	None	**1.000**	0.000	0.000	0.000	0.000
39	2.1 ⋅ 10^25^	1.0	None	**1.000**	0.000	0.000	0.000	0.000
40	3.4 ⋅ 10^24^	1.0	None	**1.000**	0.000	0.000	0.000	0.000
41	4.4 ⋅ 10^29^	0.997	None	**0.997**	0.003	0.000	0.000	0.000
42	8.4 ⋅ 10^6^	1.0	None	**1.000**	0.001	0.000	0.000	0.000
43	1047.9	0.971	None	**0.978**	0.050	0.010	0.006	0.005
44	379.7	0.859	None	**0.899**	0.303	0.008	0.002	0.000
45	6.1	0.906	None	**0.938**	0.289	0.022	0.022	0.019
46	2.3 ⋅ 10^11^	0.934	None	**0.949**	0.055	0.047	0.007	0.002
47	2.9 ⋅ 10^6^	0.989	None	**0.990**	0.016	0.011	0.009	0.008
48	3.5 ⋅ 10^24^	1.0	None	**1.000**	0.000	0.000	0.000	0.000
49	376.0	1.0	None	**0.982**	0.251	0.000	0.000	0.000
50	34.0	1.0	None	**1.000**	0.029	0.000	0.000	0.000
51	68.5	0.997	None	**0.997**	0.422	0.026	0.013	0.008
52	2.2 ⋅ 10^7^	1.0	None	**1.000**	0.000	0.000	0.000	0.000
53	2.9 ⋅ 10^6^	0.985	None	**0.963**	0.246	0.015	0.000	0.000
54	1.0	1.0	36	**1.000**	1.000	0.128	0.128	0.000
55	1.8 ⋅ 10^21^	1.0	None	**1.000**	0.128	0.128	0.128	0.000
56	3.4 ⋅ 10^9^	0.995	None	**0.997**	0.128	0.128	0.128	0.004
57	597.8	1.0	None	**1.000**	0.000	0.000	0.000	0.000
58	1.0 ⋅ 10^4^	0.896	None	**0.918**	0.166	0.031	0.018	0.004
59	25.7	0.974	None	**0.980**	0.158	0.006	0.005	0.002
60	8.6 ⋅ 10^12^	1.0	None	**1.000**	0.002	0.000	0.000	0.000
61	4.6 ⋅ 10^10^	1.0	None	**1.000**	0.000	0.000	0.000	0.000
62	43.0	0.994	None	**0.995**	0.425	0.027	0.005	0.001
63	3.0 ⋅ 10^17^	1.0	None	**1.000**	0.000	0.000	0.000	0.000
64	1.3 ⋅ 10^22^	1.0	None	**1.000**	0.000	0.000	0.000	0.000
65	1.6 ⋅ 10^14^	0.999	None	**0.999**	0.013	0.004	0.001	0.001
66	1.0	0.966	15,67,69,75	**0.973**	0.528	0.518	0.513	0.353
67	1.0	0.996	15,66,69,75	**0.997**	0.697	0.652	0.522	0.512
68	9.3 ⋅ 10^92^	0.943	None	**0.946**	0.025	0.015	0.014	0.012
69	1.0	0.991	15,66,67,75	**0.993**	0.699	0.361	0.352	0.352
70	5.3 ⋅ 10^6^	0.999	None	**0.999**	0.001	0.000	0.000	0.000
71	1.4 ⋅ 10^10^	0.995	None	**0.996**	0.004	0.002	0.002	0.002
72	1.1 ⋅ 10^16^	1.0	None	**1.000**	0.001	0.000	0.000	0.000
73	3.0 ⋅ 10^17^	1.0	None	**1.000**	0.000	0.000	0.000	0.000
74	1.1 ⋅ 10^11^	1.0	None	**1.000**	0.000	0.000	0.000	0.000
75	1.0	0.997	15,66,67,69	**0.997**	0.651	0.516	0.511	0.351
76	6.7 ⋅ 10^16^	1.0	None	**1.000**	0.000	0.000	0.000	0.000
77	5.6 ⋅ 10^15^	1.0	None	**1.000**	0.000	0.000	0.000	0.000
78	5.3 ⋅ 10^27^	1.0	None	**1.000**	0.000	0.000	0.000	0.000
79	5.2 ⋅ 10^14^	1.0	None	**1.000**	0.076	0.076	0.072	0.072

This table summarizes the likelihood of click sequences generated using a given strategy and the average relative likelihoods of the generated click sequences under the top 5 strategies on the 5-step transfer task

As you can see from the position of the bolded average likelihood ratios in Table 6, the true strategy was always the most likely explanation, on average. Furthermore, for all strategies except for one, our method’s inferences were correct most of the time. Concretely, as shown in the third column, the proportion of correct inferences ranged from 64.3% to 100% with an average of 91.8% with the exception of Strategy 13, for which the proportion of correct inferences was only 14.6*%*.

Except for Strategy 13, all strategies also had acceptable average likelihood ratios of at least 0.720. Strategy 13, which explores two extra final outcomes after uncovering a positive final outcome, had an average likelihood ratio of only 0.410. This strategy was most often confused with Strategy 9, which explores one more final outcome after uncovering a final outcome with a value greater than or equal to the second largest value of the reward distribution. To see if these strategies are distinguishable on other environments, we performed the same analysis on the 5-step transfer task. The results of this analysis are reported in Table 7. We see that Strategy 13 is identifiable on the transfer task while all other strategies remain identifiable in terms of having high relative likelihoods on average.

The LR_1,2_ ratio in the second column of Tables 6 and 7 shows how much more likely the inferred strategy tends to be compared the second most likely strategy. And the fourth column (“Similar Strategies”) shows what the second most likely strategies tend to be. We set our criterion for what it means for the computational microscope to be highly confident about the inferred strategy to LR_1,2_ = 1.5 meaning that the inferred strategy is at least 1.5 times as likely as the second most likely explanation. We found that on the 3-step increasing variance environment, this was the case for 82*%* of the strategies and on the transfer task this proportion was 80*%*, and the proportion of strategies for which this was the case for at least one of the two environments was 92*%*. The only exceptions are Strategies 6, 14, 17, 36, 67 and 69. Those strategies jointly accounted for 44*%* of all human click sequences. This is primarily because this set includes the optimal strategy and many strategies that are very similar to it. Yet, even though those strategies are very similar, the third columns of Tables 6 and 7 show that our computational microscope can nevertheless correctly identify them most of the time. This suggests that our computational microscope is usually able to confidently infer which strategy best explains a given click sequence.

We also investigated how many other alternative answers there are when the inferred strategy is less than 1.5 times as likely as the most likely alternative. In the 3-step increasing variance environment, 86% of the inferences that our method was not highly confident about (i.e., LR_1,2_ < 1.5) had only one alternative explanation, 7*%* had 2 possible alternative explanations, and 7% had 3 possible alternative explanations. Thus the median number of alternative explanations for inferences that our method is not highly confident about was only 1. In 57*%* of the cases the alternative explanations were other strategies of the same type as the inferred strategy and in the other 43% of the cases there was at most one alternative strategy type. For the 5-step increasing variance environment, the median number of alternative explanations for such inferences was 3; in 21.5% of the cases there was only 1 alternative explanation, in 21.5*%* of the cases there were 2 alternative explanations, and in 57% of the cases there were 3 alternative explanations. In 29*%* of the cases all alternative strategies were of the same type as the inferred strategy; in 42% of the cases they included one additional strategy type, and in 29% of the cases they included two or more alternative strategy types.

Overall, our findings suggest that most of the strategies are highly identifiable in at least one of the environments and that even when our method is uncertain about the strategy, there is only a small number of alternative explanations and those alternative explanations often instantiate the same strategy type.

### A.6 Features

The strategies described in section 1 were implemented using the 51 features described below. The features are grouped using the decision-making factor they represent. These features are defined in terms of the nodes in the Mouselab-MDP paradigm. All the features take a belief state, computation pair as input. We define the considered node to be the entity whose value is found out after clicking. The value of all the features is 0 for termination unless otherwise specified.

#### A.6.1 Mental effort avoidance

**Feature 1: “Termination Constant”:** The value of this feature is 1 for all clicks and 0 for the termination operation in all belief states.

#### A.6.2 Model-based metareasoning features

These features capture uncertainty about the values of the unobserved nodes. Uncertainty is defined as the standard deviation of the values of the distribution. The following features capture uncertainty:**Feature 2: “Uncertainty”**: The value of this feature for a click in a given belief state is the uncertainty in the value of the considered node.**Feature 3: “Max Uncertainty”**: The value of this feature for a click in a given belief state is the is the maximum uncertainty in return for the current trial from all the paths that the considered node lies on.**Feature 4: “Successor Uncertainty”**: The value of this feature for a click in a given belief state is the total uncertainty in the values of all the successors of the considered node on the current trial.**Feature 5: “Trial level standard deviation”**: The value of this feature for a click is the uncertainty in the value of the considered node as estimated across all trials attempted so far by the agent.**Feature 6: “Current trial level standard deviation”**: The value of this feature for a click in a given belief state is the uncertainty in the value of nodes at the same depth as the considered node as estimated for the current trial.**Feature 7: “Does the node lie on the second most promising path?”**: The value of this feature for a click in a given belief state is 1 if the considered node lies on the path which has the second highest expected return for the current trial, and 0 otherwise.

#### A.6.3 Pavlovian Features

These features are based on greedy maximization. Pavlovian behavior is captured by the following features:**Feature 8: “Best expected value”**: The value of this feature for a click in a given belief state is the best expected return for a path in the current trial among all the paths that the considered node lies on.**Feature 9: “Best largest value”**: The value of this feature for a click in a given belief state is the maximum value observed among all the paths that the considered node lies on.**Feature 10: “Does the node lie on the most promising path?”**: The value of this feature for a click in a given belief state is 1 if the considered node lies on the path with the highest expected return for the current trial, and 0 otherwise.**Feature 11: “Value of the max expected return”**: The value of this feature for all clicks in a given belief state is the maximum expected return from all paths in the current trial.**Feature 12: “Does a successor node have a maximum value?”**: The value of this feature for a click in a given belief state is 1 if any of the considered node’s observed successors in the current trial has a value which is the maximum possible value for the reward distribution, and 0 otherwise.**Feature 13: “Maximum value of a successor”**: The value of this feature for a click in a given belief state is the maximum value that has been observed among all the successors of the considered node in the current trial.**Feature 14: “Maximum value of an immediate successor”**: The value of this feature for a click in a given belief state is the maximum value that has been observed among all the immediate successors of the considered node in the current trial.**Feature 15: “Value of the parent node”**: The value of this feature for a click in a given belief state is the value of the considered node’s parent if the parent node has been observed, and 0 otherwise.

##### Pruning features

These features are designed to capture the idea of pruning branches (Huys et al., 2012). The value for these features for all clicks is -1 if the maximum expected loss that can be incurred in the current belief state is worse than the pruning threshold and 0 otherwise. We consider features with different pruning thresholds: -48, -24, -8 and 0 (features 16-19). In addition, we consider the following features:**Feature 20: “Soft Pruning”**: The value of this feature for a clicks is the maximum expected loss that can be incurred in a given belief state from all paths that the considered node lies on.**Feature 21: “Is the previous observed node a successor and has negative value”**: The value of this feature for a click in a given belief state is 1 if the last observed node in the current trial is a child of the considered node and has a negative value, and 0 otherwise.

#### A.6.4 Satisficing and stoppping features

##### Satisficing features

These features determine when the planning satisfices (Simon, 1956). The value for these features is -1 for termination if the maximum expected return for the current trial is greater than the satisficing threshold. We consider features with different satisficing thresholds: 0, 8, 16, 24, 32, 40 and 48 (features 22-28). In addition, we consider the following 2 features:**Feature 29: “Soft Satisficing”**: The value of this feature for all clicks in a given belief state is the maximum return that can be expected on the current trial from all paths that the considered node lies on.

##### Stopping Criteria

These features have same value for all the clicks and a different value for termination.**Feature 30: “Are all max paths observed?”**: The value of this feature is -1 for all clicks and 0 for termination action in all belief states if all the paths path leading to a final outcome, which has the maximum value among the observed final outcomes, has been observed in the current trial and 0 otherwise.**Feature 31: “Is a max path observed?”**: The value of this feature is -1 for all clicks in all belief states if any path leading to the node, which has the maximum value possible for the reward distribution, has been observed in the current trial and 0 otherwise.**Feature 32: “Is a positive node observed?”**: The value of this feature is -1 for all clicks in all belief states if a node with a positive value has been observed in the current trial and 0 otherwise.**Feature 33: “Is the previous observed node maximal?”**: The value of this feature is -1 for all clicks if the last observed node in the current trial has the maximum value possible for the reward distribution and 0 otherwise.**Feature 34: “Is a complete path observed?”**: The value of this feature is -1 for all nodes in all belief states if at least one path has been completely observed from immediate outcome to final outcome, and 0 otherwise.**Feature 35: “All final outcomes observed?”**: The value of this feature is -1 for all clicks in all belief states if all final outcomes have been observed in the current trial and 0 otherwise.**Feature 36: “Are all immediate outcomes observed?”**: The value of this feature is -1 for all clicks in all belief states if all immediate outcomes have been observed in the current trial and 0 otherwise.**Feature 37: “Are final outcomes of positive immediate outcomes observed?”**: The value of this feature is -1 for all clicks in all belief states if all the final outcomes that can be reached from the positive observed immediate outcomes have been observed, and 0 otherwise.

#### A.6.5 Model-free values and heuristics features

##### Relational features

The values of these features for a considered node are dependent on its neighboring nodes.**Feature 38: “Ancestor count”**: The value of this feature for a click in a given belief state is the number of ancestors of the considered node that have been observed in the current trial.**Feature 39: “Depth Count”**: The value of this feature for a click in a given belief state is the number of times that any node at the same depth as the considered node has been observed in the current trial.**Feature 40: “Is the node a final outcome and has a positive ancestor?”**: The value of this feature for a click in a given belief state is 1 if the considered node is a final outcome and it has an observed ancestor with a positive value in the current trial and 0 otherwise.**Feature 41: “Immediate successor count”**: The value of this feature for a click in a given belief state is the number of children of the considered node that have been observed in the current trial.**Feature 42: “Is parent observed?”**: The value of this feature for a click in a given belief state is 1 if the parent node of the considered node has been observed, and 0 otherwise.**Feature 43: “Successor Count”**: The value of this feature for a click in a given belief state is the number of observed successors of the considered node for the current trial.**Feature 44: “Squared Successor Count”**: The value of this feature for a click in a given belief state is the square of the number of observed successors of the considered node for the current trial.**Feature 45: “Siblings Count”**: The value of this feature for a click in a given belief state is the number of siblings of the considered node that have been observed in the current trial.**Feature 46: “Minimum number of observed nodes on branch”**: The value of this feature for a click in a given belief state is the minimum number of nodes observed on all the branches containing the considered node.**Feature 47: ”Is the previous observed node a successor?”**: The value of this feature for a click in a given belief state is 1 if the last observed node in the current trial is one of the successors of the considered node, and 0 otherwise.

##### Structural features

The values of these features are dependent no the task structure.**Feature 48: “Depth”**: The value of this feature for a click in a given belief state is the distance of the considered node from the starting position.**Feature 49: “Is the node an immediate outcome?”**: The value of this feature for a click in a given belief state is 1 if the considered node in an immediate outcome and 0 otherwise.**Feature 50: “Is the node a final outcome?”**:The value of this feature for a click in a given belief state is 1 if the considered node is a final outcome and 0 otherwise.**Feature 51: “Observed height”**: The value of this feature for a click in a given belief state is the length of the maximum observed path to a final outcome starting from the considered node.

## Footnotes

1 Those strategies may be composed of building blocks that are reused in multiple strategies but we do not explicitly model this hierarchical structure.

2 Here and throughout the manuscript, we always report the results of a Bayesian hypothesis testing along with their frequentist equivalents. We performed Bayesian hypothesis testing using the BayesFactor package in R (Morey & Rouder, 2018) for all tests except the Wilcoxon test for which we used JASP (JASP Team, 2021). We report the results of each Bayesian hypothesis test in terms of the Bayes factor in favor of the alternative hypothesis. *B**F* = *x* means that the alternative hypothesis is *x* times as likely to be true as the null hypothesis.
